# Generalized Linear Model with Elastic Net Regularization and Convolutional Neural Network for Evaluating Aphanomyces Root Rot Severity in Lentil

**DOI:** 10.34133/2020/2393062

**Published:** 2020-11-13

**Authors:** Afef Marzougui, Yu Ma, Rebecca J. McGee, Lav R. Khot, Sindhuja Sankaran

**Affiliations:** ^1^Department of Biological Systems Engineering, Washington State University, Pullman, WA, USA; ^2^Department of Horticulture, Washington State University, Pullman, WA, USA; ^3^United States Department of Agriculture-Agricultural Research Service, Grain Legume Genetics and Physiology Research Unit, Washington State University, Pullman, WA, USA

## Abstract

Phenomics technologies allow quantitative assessment of phenotypes across a larger number of plant genotypes compared to traditional phenotyping approaches. The utilization of such technologies has enabled the generation of multidimensional plant traits creating big datasets. However, to harness the power of phenomics technologies, more sophisticated data analysis methods are required. In this study, Aphanomyces root rot (ARR) resistance in 547 lentil accessions and lines was evaluated using Red-Green-Blue (RGB) images of roots. We created a dataset of 6,460 root images that were annotated by a plant breeder based on the disease severity. Two approaches, generalized linear model with elastic net regularization (EN) and convolutional neural network (CNN), were developed to classify disease resistance categories into three classes: resistant, partially resistant, and susceptible. The results indicated that the selected image features using EN models were able to classify three disease categories with an accuracy of up to 0.91 ± 0.004 (0.96 ± 0.005 resistant, 0.82 ± 0.009 partially resistant, and 0.92 ± 0.007 susceptible) compared to CNN with an accuracy of about 0.84 ± 0.009 (0.96 ± 0.008 resistant, 0.68 ± 0.026 partially resistant, and 0.83 ± 0.015 susceptible). The resistant class was accurately detected using both classification methods. However, partially resistant class was challenging to detect as the features (data) of the partially resistant class often overlapped with those of resistant and susceptible classes. Collectively, the findings provided insights on the use of phenomics techniques and machine learning approaches to provide quantitative measures of ARR resistance in lentil.

## 1. Introduction

Crop phenotyping refers to a key process in crop improvement programs, associated with the evaluation of expressed plant traits as a result of interaction between the genotype and the environment. Phenotyping can be cumbersome [1] due to the low throughput and subjectivity associated with conventional techniques. Such limitations require the development of phenomics technologies, which often refers to technology-assisted acquisition of multidimensional phenotypic data at cellular, organ, plant, or population levels [2]. It is anticipated that the advancements in phenomics can enable the evaluation of large-scale breeding trials, nondestructively, automatically, and at a high spatial-temporal resolution compared to conventional methods.

Phenomics tools facilitate large-scale screening of many traits [3] through advances in sensor technologies [4–8]. Such methods generate “big data” that need to be analyzed to extract meaningful digital traits, thus, involving new approaches for data analysis [9–11]. For instance, machine learning (ML) approaches have been discussed in multiple studies. It has been demonstrated that both statistical and ML approaches could be employed efficiently to discern patterns from the collected phenotypic data [9, 12]. One of the advantages of employing ML tools is that it allows the evaluation of combinations of these traits instead of evaluating plant traits individually. The ability to explain a particular biological pattern through data-driven approaches—such as disease resistance and agronomic performances —can help plant breeders, plant pathologists, and physiologists in their decision-making [9].

Recent studies have also demonstrated the applicability of sensing data integrated ML tools in phenotyping biotic stress. These ML applications can be summarized into four primary folds: identification/detection, classification, quantification/estimation, and prediction [9]. However, feature extraction and/or engineering remain a significant bottleneck to implement such techniques. It requires domain expertise to derive and extract digital traits that characterize a particular trait or trend. In recent years, with the improvement of computational power and the availability of Graphic Processing Units (GPU), deep learning (DL)—a subfield of ML—has been widely used in the machine vision community as a tool for feature extraction and decision-making and has gained prominence in phenomics [10, 11, 13]. For instance, convolutional neural networks (CNN) have been used to detect plant diseases [14–17]. However, the underlying process of such DL techniques remains a challenging aspect to understand and interpret the obtained results. Therefore, few studies have started to focus on the explanation of these “black boxes” (process of inference/decision) associated with DL architecture [14, 18]. For example, approaches such as top-K high-resolution profile maps were proposed to visualize the predictions associated with DL-based detection of foliar stress symptoms in soybean to better understand the model application [14]. Similarly, neuron-wise and layer-wise visualization methods were also applied using a CNN to better understand the model authenticity associated with soybean leaf stress detection. Color and texture lesions were found to be associated with the CNN decision-making process [18], thus providing a connection between the disciplinary knowledge domain and ML tools.

In this study, Aphanomyces root rot (ARR) resistance in lentil was evaluated with Red-Green-Blue (RGB) images. ARR disease is considered a significant limitation in lentil and pea production, which can result in severe economic losses [19]. The absence of disease resistance in commercial cultivars has led to efforts towards development of lentil cultivars with better disease resistance through breeding and genetics programs. In an effort to assist in the process of phenotyping, in our previous work [20], we evaluated the potential of RGB and hyperspectral image features extracted from lentil shoots/roots integrated with an elastic net regression model for disease class prediction. We found that the RGB features (color, texture, geometry) associated with the root images showed promising results. Given the potential benefits of DL tools, in this study, we built and compared two approaches, generalized linear model with elastic net regularization (EN) and deep learning (CNN) models, to classify ARR disease severity using lentil root images into three (resistant, partially resistant, and susceptible) classes with a larger dataset.

## 2. Materials and Methods

### 2.1. Aphanomyces Root Rot Disease Image Dataset

**Figure 1:**
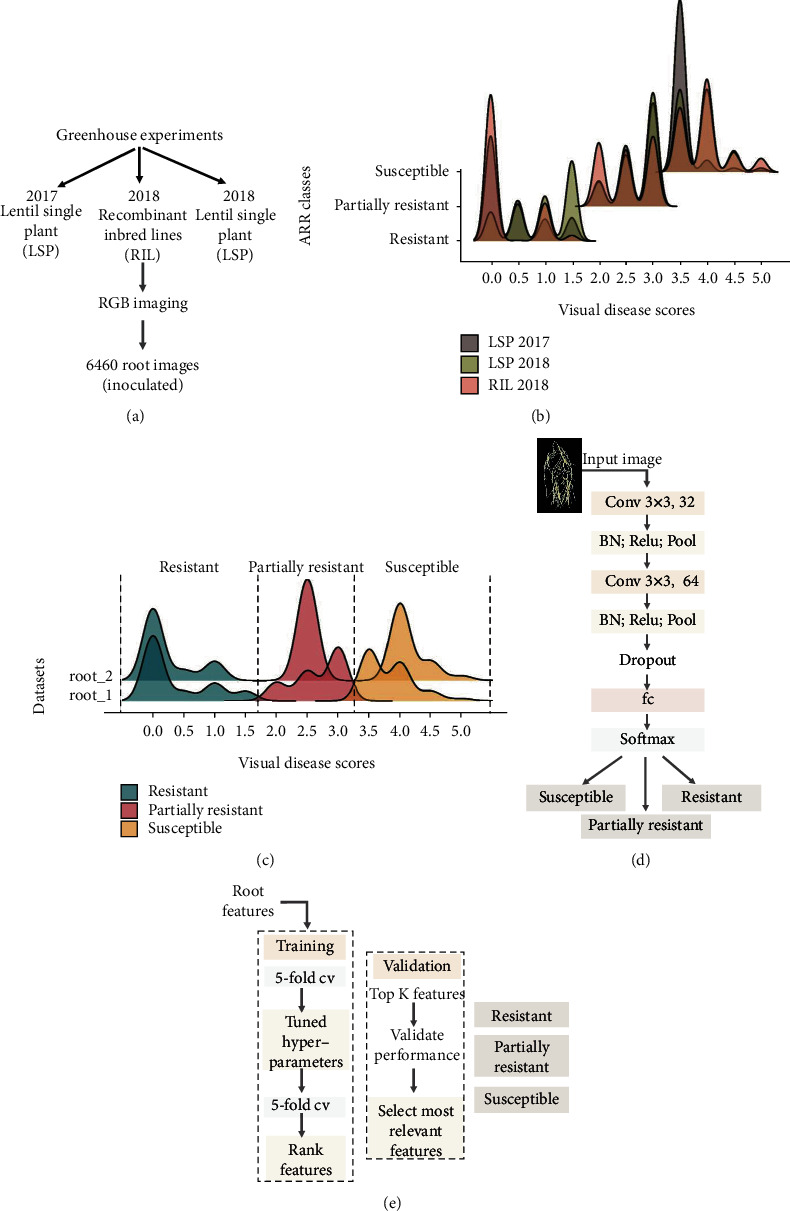
Data analysis approaches: training and optimization. (a) Imagery datasets, (b) distribution of ARR visual disease scores and ARR disease classes within experiments, (c) distribution of ARR visual disease scores and ARR disease classes within root_1 dataset (*n* = 6,460images) and root_2 dataset (*n* = 3,275images), (d) CNN architecture, and (e) generalized mixed model with EN regularization optimization and feature selection. Conv: convolutional layer; BN: batch normalization layer; Relu: rectified linear unit layer; Pool: max pooling layer; Dropout: dropout layer; fc: fully connected layer; softmax: softmax layer; cv: cross-validation.

RGB images of lentil plants (roots) were captured from three separate experiments grown in controlled environmental conditions. The first experiment, conducted in June 2017, included 353 lentil accessions from the USDA lentil single plant-derived (LSP) collection and was planted using a split-plot design with five replicates. The second experiment was conducted in February 2018. This experiment used a biparental recombinant inbred line (RIL) population. There were 195 RILs planted in a completely randomized design with three replicates and three samples for each replicate. The third experiment was conducted in November 2018 and consisted of 334 lentil accessions from the LSP grown in a randomized complete block design with ten replicates. In all experiments, plants were grown in a greenhouse with a day temperature of 25°C, night temperature of 23°C, and photoperiod of 16 h. Details for inoculum preparation and inoculation of plant material are described in our previous studies [20, 21]. The procedure for RGB image capture is also described in our previous work [20]. This study focused on features extracted from root images. Prior to analysis, all images were labeled based on an expert's visual disease scores, a standard phenotyping protocol adapted from the literature [22]. Roots were screened for the percentage of brown discoloration and hypocotyl softness, giving them a visual disease score ranging from 0.0 to 5.0 (*Supplementary Materials Table*S1). Images were preprocessed by removing the background (pixels that do not belong to roots, as described in [20]) and were then divided into three classes based on the visual scores: resistant (score of 0.0 to 1.5), partially resistant (score of 2.0 to 3.0), and susceptible (score of 3.5 to 5.0). The final dataset includes 6,460 root images, of which 1,428 were scored as resistant; 2,529 as partially resistant; and 2,503 as susceptible (Figures 1(a) and 1(b)).

### 2.2. Feature Extraction from Root Images

The extraction and selection of relevant features often govern the performance of ML models. In this work, CNN and selected RGB features combined with generalized mixed model with elastic net regularization were independently employed to classify ARR disease severity in lentil root into three classes. All root images were resized to a pixel size of 227 × 227 prior to analysis. Two main approaches were utilized. First, the performance of both models was evaluated using the complete dataset of inoculated roots (root_1dataset = 6,460 RGB images). In the second approach, the model performances were evaluated using a reduced dataset, in which images at the border of each class (i.e., visualscores = 1.5, 2.0, 3.0, and 3.5) were removed (root_2dataset = 3,275 RGB images). Details about the class distributions are presented in Figure 1(c). Both image datasets were randomly divided 10 times (Table 1)—based on their label class—into training, validation, and testing (splitting ratio of 80/10/10) using 10 different random seeds.

#### 2.2.1. CNN Model Architecture

CNN is a multilayer neural network that is often used in machine vision to analyze imagery datasets for classification or object detection tasks. It is a supervised learning method that enables the extraction of features and the training of a classifier within the same network [16]. In this study, a small CNN architecture was used to prevent model overfitting [23] (Figure 1(d)). The input images were zero-center normalized. A total of 32 kernels with a size of 3 × 3 and a stride size of 1 were used for convolving the input of three channel RGB images. The same convolutional kernel size and stride were used in the second convolutional layer, but the number of filters was increased to 64. Each convolutional layer was followed by a batch normalization (BN) and an activation function (ReLU = rectified linear unit). In addition, a 2 × 2 max-pooling layer was applied on the output of each convolutional layer. Dropout, with a probability of 0.20, was performed before the fully connected layer to prevent overfitting [17, 24]. The output of the fully connected layer was fed to a softmax layer, which is a linear classifier. Additional details regarding CNN training are presented in Table 2.

The CNN model was implemented using MATLAB® Deep Learning Toolbox (2019a, The MathWorks, Natick, MA, USA) and was trained on a single GPU (NVIDIA GeForce GTX 1080; 8 GB memory) with CUDA 10.0. The CNN was optimized initially on root_1 dataset (trained using 5,167 images with an additional 647 images for validation) by selecting the number of layers, number and size of filters, solver type, learning rate and learning schedule, and batch-size. The same selected parameters were evaluated on root_2 dataset (trained using 2,620 images with an additional 328 images for validation), in an assumption that root_2 dataset will reduce the noise resulting from boundary miscategorization. CNN performances were monitored by checking the classification accuracy and the cross-entropy loss of both the training (minibatch data) and the validation datasets.

#### 2.2.2. Generalized Linear Model with EN Regularization

The image features were extracted as described in our previous work [20]. The EN model was trained using 78 root features (*Supplementary Materials Table*S2). Elastic net is a regularization technique, combining least absolute shrinkage and selection operator (LASSO) and ridge regression. LASSO utilizes L1 regularization as a penalty method, and ridge regression utilizes L2 regularization [25]. The penalty parameters (*α* and *λ*) were firstly tuned on the training set through a 5-fold cross-validation (Figure 1(e)). The selected parameters were used to train the model for a second time, and the list of nonzero contributing features obtained from each run of a 5-fold cross-validation was saved. For a robust feature selection, a stability criteria approach was developed, aiming at retaining top K ranked features that resulted in the best performance of the EN model. For this step, features were ranked in a decreasing order based on their importance score (scaled variable importance scores from 100 to ~0). We iterated through the ordered lists of features 14 times (toprankedfeatures = 10, 15, 20, 25, 30, 35, 40, 45, 50, 55, 60, 65, 70, and 78) and trained the EN model using the obtained K features. This time, the results were validated using the validation datasets. At the end of this step, for each list of K features, the corresponding overall accuracy and by class performances were saved. The selection of K features was a trade-off between reasonable *F*1 by class scores and overall accuracy. We implemented the EN model using the *glmnet* method in the “caret” package [26] in R (http://www.r-project.org/; release 3.6.0). The data were scaled and centered to zero (preprocess option in caret). Each time the EN model was trained, we used a grid-search to tune both *α* and *λ*.

### 2.3. Evaluation Metrics

Accuracy (Eq. (1)), precision (Eq. (2)), recall (Eq. (3)), and *F*1 score (Eq. (4)) were used as multiclass performance metrics to evaluate the performance of classification tasks.

(1) $$\begin{array}{c} \text{Accuracy}=\frac{\text{True Positives}+\text{True Negatives}}{\text{True Positives}+\text{True Negatives}+\text{False Positives}+\text{False Negatives}} \end{array}$$

(2) $$\begin{array}{c} \text{Precision}=\frac{\text{True Positives}}{\text{True Positives}+\text{False Positives}} \end{array}$$

(3) $$\begin{array}{c} \text{Recall}\left(\text{or Sensitivity}\right)=\frac{\text{True Positives}}{\text{True Postives}+\text{False Negatives}} \end{array}$$

(4) $$\begin{array}{c} F1\text{score}=\frac{2\times \text{Precision}\times \text{Recall}}{\text{Precision}+\text{Recall}} \end{array}$$

Finally, nonmetric multidimensional scaling (nMDS) was employed to visualize the CNN features (the output of fully connected layer as logit values) and the RGB selected features from the EN model. The purpose of this method is to map distances (similarities or dissimilarities) between samples into lower dimensions. The number of dimensions was selected based on a stress-dimension plot (*Supplementary Materials Figure*S1). The nMDS ordination was performed using the “isoMDS” function implemented in the R package “MASS” [27]. Additionally, a nonparametric Spearman correlation analysis was conducted using “cor” function implemented in R to assess the similarities between the extracted RGB features and visual disease scores.

## 3. Results

### 3.1. Model Performance and Hyper-Parameter Tuning

**Figure 2:**
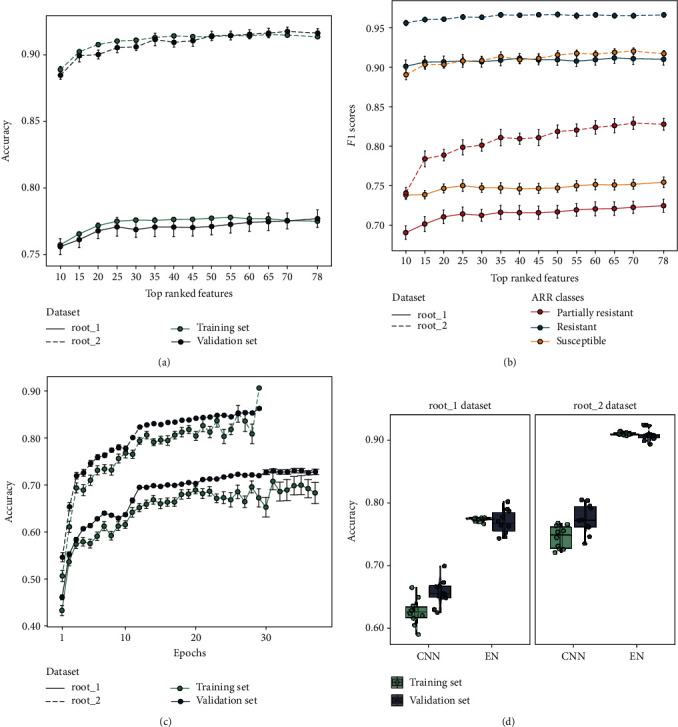
Training and validation performances of both EN and CNN trained on root_1 and root_2 datasets: (a) variation of training and validation accuracies of EN with the ranked RGB features averaged across the 10 random runs, (b) variation of by-class *F*1 scores of EN with the ranked RGB features averaged across the 10 random runs, (c) training and validation accuracies of CNN averaged across the 10 random runs, and (d) boxplot of training and validation accuracies of EN and CNN; each point represented a random run. The error bars corresponded to standard error from the 10 random runs.

The performance of the EN model based on selected RGB features varied depending on the dataset type. The model performed better when trained on root_2 dataset (mean validation accuracy = 0.91 ± 0.004) compared to root_1 dataset (mean validation accuracy = 0.77 ± 0.006). In general, the classification accuracy stabilized after including more than the top 25 ranked features (Figure 2(a)). For the root_1 dataset, a total number of 60 features resulted in a maximum validation accuracy of 0.82, while for root_2 dataset, a total number of 35 features gave an accuracy of 0.94 (Figures 2(a) and 2(d); *Supplementary Materials Table*S3, *Figure*S2).

The *F*1 score of the three ARR classes—with exception of partially resistant class in root_2 dataset—started to stabilize after including more than the top 25 ranked features (Figure 2(b)). The resistant class had the highest score (mean*F*1score = 0.91 ± 0.002 and 0.96 ± 0.001 for root_1 and root_2 datasets, respectively), followed by the susceptible class (mean*F*1score = 0.75 ± 0.002 and 0.91 ± 0.001 for root_1 and root_2, respectively) and the partially resistant class (mean*F*1score = 0.71 ± 0.002 and 0.81 ± 0.003 for root_1 and root_2, respectively). Interestingly, the number of top ranked features did not seem to affect the *F*1 score of the resistant class, whether the resistant class was the minority (root_1 dataset) or the dominant class (root_2 dataset).

During validation of CNN model (Figure 2(c)), trained on root_1 dataset, the model stabilized with a mean accuracy of 0.66 ± 0.001 (epochs = 37, meanvalidationloss = 0.89 ± 0.008). The CNN model, trained on root_2 dataset, resulted in a higher validation accuracy of 0.78 ± 0.002 (epochs = 29, meanvalidationloss = 0.72 ± 0.010). See *Supplementary Materials Table*S4 for detailed CNN performances.

### 3.2. Classification of ARR Severity

**Figure 3:**
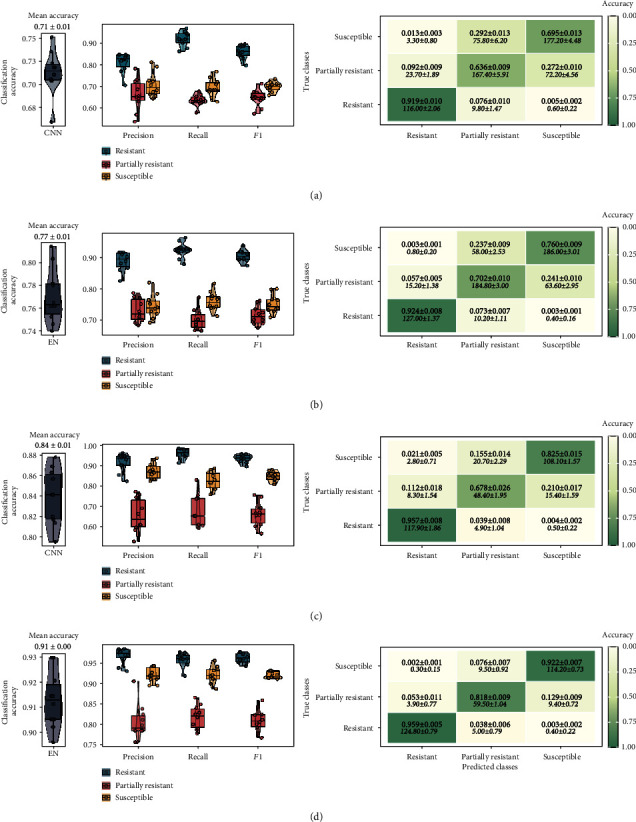
Classification model performances on test datasets averaged across the 10 random runs: (a) test results of CNN trained on root_1, (b) test results of EN trained on root_1, (c) test results of CNN trained on root_2, and (d) test results of EN trained on root_2. Figures from left to right: overall test accuracy (each point represented a random run), variation of precision, recall, and *F*1 scores by ARR class (each point represented a random run) and normalized confusion matrix (numbers in italic represented number of samples before normalization ± standarderror).

Both models were evaluated on the same test datasets (root_1dataset = 646 and root_2dataset = 327). The overall test accuracy differed between datasets used for training. The root_1 dataset, which combined root images from the three experiments (*n* = 6,460), resulted in a test accuracy of 0.71 ± 0.008 using CNN model and 0.77 ± 0.008 using EN model (Figures 3(a) and 3(b)). The reduction of the dataset, by removing the border-scored root samples (*n* = 3,275), increased the performance of both models (meantestaccuracy = 0.84 ± 0.009 and 0.91 ± 0.004 for CNN and EN, respectively) (Figures 3(c) and 3(d)).

Lentil root samples were successfully classified as resistant either using root_1 dataset (meanaccuracy = 0.92 ± 0.010 and 0.92 ± 0.008 for CNN and EN, respectively) or root_2 dataset (accuracy = 0.96 ± 0.008 and 0.96 ± 0.005 for CNN and EN, respectively). On the other hand, the root samples were successfully classified as susceptible only when using root_2 dataset (accuracy = 0.83 ± 0.015 and 0.92 ± 0.007 for CNN and EN, respectively). Both models did not perform well in distinguishing the partially resistant class. The per-class accuracy ranged between 0.64 ± 0.009 and 0.70 ± 0.010 for CNN and EN, respectively, even when the partially resistant class was the dominant class in the root_1 dataset (39.15% of the dataset). Using the root_2 dataset, the accuracy increased to 0.82 ± 0.009 for the EN; however, it slightly increased to 0.68 ± 0.026 in the case of CNN.

Sensitivity (or recall) and precision are additional metrics that can be used to understand the model performances per class (Figure 3). Both CNN and EN were able to recognize the truly resistant class (sensitivity = 0.92 ± 0.010 and 0.92 ± 0.008 for root_1 and 0.96 ± 0.008 and 0.96 ± 0.005 for root_2, with CNN and EN, respectively), indicating a low false negative rate (true class is resistant, but the root sample was classified as partially resistant or susceptible). Furthermore, the high precision rate of the resistant class (precision = 0.81 ± 0.014 and 0.89 ± 0.010 for root_1 and 0.91 ± 0.014 and 0.97 ± 0.006 for root_2, with CNN and EN, respectively) indicates a low false positive rate for this class (true class is partially resistant or susceptible, but the sample was classified as resistant).

### 3.3. Image Features Associated with ARR Resistance

**Figure 4:**
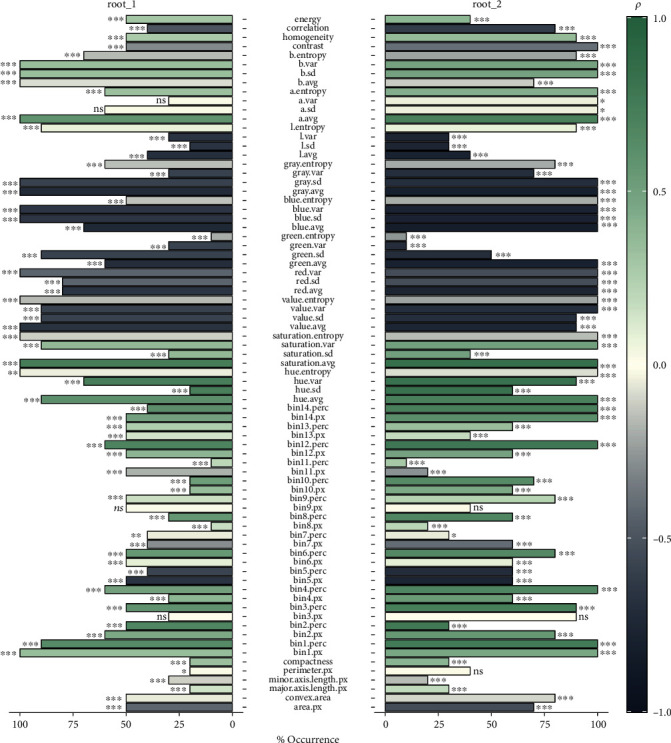
Spearman correlation analysis of the model-selected final RGB features—selected using EN—and visual disease scores (*n* = 6,460 and 3,275, for root_1 and root_2, respectively). The barplot represented the percentage of occurrence across the 10 random runs, and the color intensity represented the Spearman correlation coefficient, *ρ*. The levels of *p* value are as follows: ns: nonsignificant: *p* ≥ 0.05; ^∗^0.001 < *p* < 0.05; ^∗∗^0.0001 < *p* ≤ 0.001; ^∗∗∗^*p* ≤ 0.0001.

The correlation analysis of the extracted RGB features—selected using EN—revealed highly significant association with the visual disease scores (Spearman correlation coefficient, −0.73 ≤ *ρ* ≤ 0.74 and −0.82 ≤ *ρ* ≤ 0.84, *p* value < 0.05, for root_1 and root_2, respectively, Figure 4). A total of 15 (19.5% of total number of features) features were frequently selected across the 10 random runs for root_1 dataset, while almost double that number (39.0% of total number of features) was observed for root_2 dataset. All these features captured the color-related properties of the studied root images.

**Figure 5:**
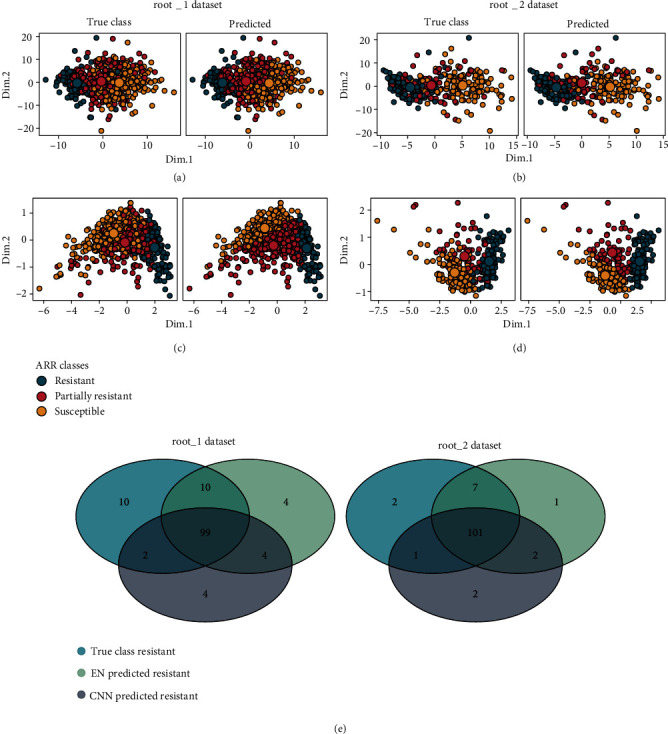
nMDS ordinations of test set (randomrun = 7): (a) RGB features selected using EN for root_1 dataset, (b) RGB features selected using EN for root_2 dataset, (c) FC features extracted from CNN for root_1 dataset, and (d) FC features extracted from CNN for root_2 dataset. (e) Venn diagram of resistant class classification averaged across the 10 random runs.

The patterns of nMDS ordinations of the final image features (fully connected/FC features from CNN and RGB selected features from EN, Figures 5(a)–5(d)) suggested that resistant and susceptible classes, whether using the annotation from ground-truth (true class) or predicted class (EN and CNN), were clustered into two separate groups. To a lesser extent, the partially resistant class clearly overlapped with both resistant and susceptible, which could explain the high rate of misclassification of this particular group.

The accessions/lines identified as resistant using both models are summarized in the Venn diagram in Figure 5(e). Most resistant accessions/lines (true class) were commonly identified as resistant using both models, with deviation of 6-8 accessions/lines. Noteworthy, in case of false positives, both models tended to classify partially resistant accessions/lines as resistant, and few susceptible accessions/lines were classified as resistant.

## 4. Discussion

Imaging technologies have enabled the quantification of plant disease resistance and have provided plant breeders with an efficient alternative to support their decision-making. In this study, we focused on the classification of ARR resistance of 547 lentil accessions and lines. The evaluation was conducted using two supervised approaches. First, a more traditional approach was utilized, combining selected RGB features with generalized linear model with elastic net regularization. The extracted features included shape, color, and texture features. These features are also known, in machine vision tasks, as global or low-level image features that can be used to summarize an image in low-dimensional numerical representations [28]. In the second approach, we used a deep learning model, where CNN was developed as an end-to-end approach to classify ARR severity classes from root images. The labeled images were categorized into resistant, partially resistant, and susceptible classes according to their visual disease scores. To the best of our knowledge, this is the first study on the evaluation of ML tools and image features for root disease severity classification.

The experimental results showed that an increase in dataset size, in terms of the number of samples, does not necessarily translate into a better predictive power of developed models. The number of classes, the similarity between classes, and the variation within the same class, all play a vital role in the selection of features and model performances. One crucial aspect revealed by this study was that, regardless of the approach and the type of dataset, lentil accessions/lines were successfully classified as resistant with higher precision and recall scores compared to partially resistant and susceptible classes. Ideally, for a classification task, maximizing both precision and recall (or the ratio *F*1 score) would be set as a target to improve the classifier performance. However, it is usually challenging to maximize both metrics at the same time. In general, there is a trade-off between the two factors (precision and recall) that can be set based on the overall objective of a classification solution. For instance, if the focus is towards detecting resistant class, a model with a high recall rate will capture as many accessions/lines classified as resistant. Such a scenario may result in some false positives (partially resistant or susceptible classified as resistant). This implies that further screening stages are needed to filter out the selected accessions/lines. On the other hand, a model with high precision for the resistant class will yield less false positives but will lose the opportunity to choose some resistant accessions/lines (false negative). We believe that the balance of both metrics varies depending on the plant breeder perspectives as well as the stage of the breeding cycle.

During examination of the relationships between the ML features and the ground-truth data (ARR classes and visual scores), the results indicated that with selected features and the traditional approach, we could provide a set of low-level features as a quantitative approximation of the ARR resistance that corresponds to the ground-truth data. Although, with the CNN approach, the output features of the fully connected layer gave similar visual patterns compared to the ground-truth, the process of obtaining these features is more computationally complex than the traditional approach. Additionally, the complexity of tuning the CNN model hindered its scalability to a larger image resolution. The same tuned model failed to capture the differences between classes when the input images were rescaled to the original size (data not shown). In summary, our results suggest that unless the CNN approach would result in better performances, an extraction of low-level features coupled with another simpler model would be a practical solution for ARR resistance evaluations in lentil.

## Figures and Tables

**Table 1:** Distribution of ARR disease classes within root_1 and root_2 datasets.

Dataset	Type	Resistant	Partially resistant	Susceptible	Total
root_1	Train	1,142	2,023	2,002	6,460
	Validation	143	253	251	
	Test	143	253	250	

root_2	Train	1,034	593	993	3,275
	Validation	130	74	124	
	Test	129	74	124	

**Table 2:** CNN hyperparameters used in this study.

Solver type	Stochastic gradient descent
Initial learning rate	1 × 10^−4^
Learning rate schedule	Piecewise: decreases by factor of 0.1 every 10 epochs
Batch size	32
Momentum	0.9
Loss function	Cross entropy
L2 regularization	1 × 10^−4^

## Data Availability

Data available at: https://doi.org/10.5281/zenodo.4018168.

## Supplementary Materials

Supplementary Materials Figure S1: nonmetric multidimensional scaling scree plot. Figure S2: final RGB feature importance evaluated using EN model for root_1 and root_2. Table S1: Aphanomyces root rot visual disease scoring criteria. Table S2: list of root features extracted from RGB images. Table S3: number of selected features based on their importance scores. Table S4: CNN performance during training and validation (averaged across the 10 random runs).
