# Supplementary material for: Generalized Linear Model with Elastic Net Regularization and Convolutional Neural Network for Evaluating Aphanomyces Root Rot Severity in Lentil
Source: Plant Phenomics. 2020 Nov 13;2020:2393062. doi: 10.34133/2020/2393062 (PMC7870103; doi:10.34133/2020/2393062)
Supplement: Supplementary Materials — Figure S1: nonmetric multidimensional scaling scree plot. Figure S2: final RGB feature importance evaluated using EN model for root_1 and root_2. Table S1: Aphanomyces root rot visual disease scoring criteria. Table S2: list of root features extracted from RGB images. Table S3: number of selected features based on their importance scores. Table S4: CNN performance during training and validation (averaged across the 10 random runs). [file 2393062.f1.docx]

**Generalized Linear Model with Elastic Net Regularization and Convolutional Neural Network for Evaluating Aphanomyces Root Rot Severity in Lentil**

Afef Marzougui^1^, Yu Ma^2^, Rebecca J. McGee^3^, Lav R. Khot^1^, Sindhuja Sankaran^1*^

^1^Department of Biological Systems Engineering, Washington State University, Pullman, WA, USA

^2^Department of Horticulture, Washington State University, Pullman, WA, USA

^3^United States Department of Agriculture-Agricultural Research Service, Grain Legume Genetics and Physiology Research Unit, Washington State University, Pullman, WA

*Correspondence should be addressed to Sindhuja Sankaran; [sindhuja.sankaran@wsu.edu](mailto:sindhuja.sankaran@wsu.edu)


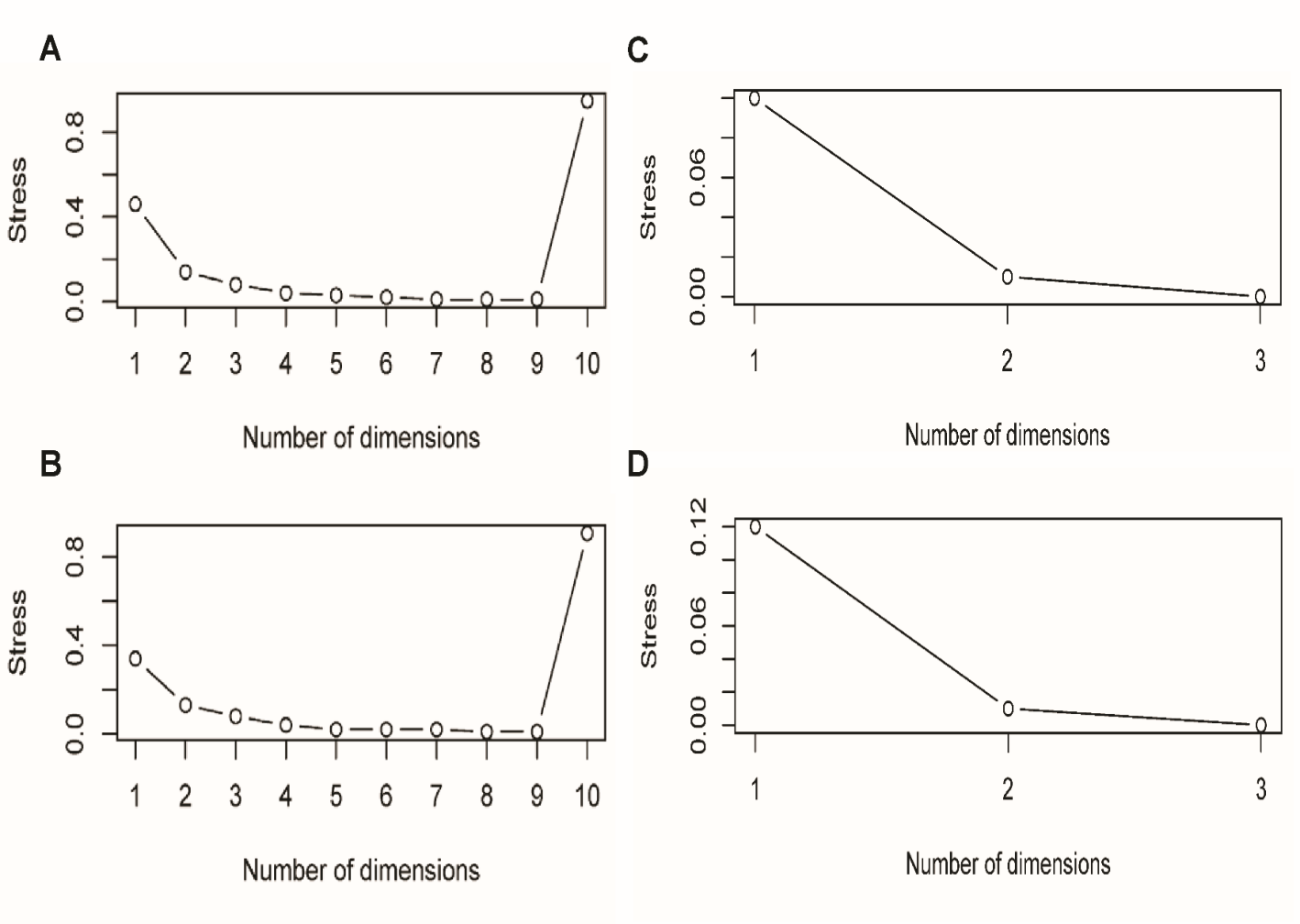


**Figure S1.** Nonmetric multidimensional scaling scree plot. The variation of stress with each dimension for: (A) RGB features from root_1 dataset (EN), (B) RGB features from root_2 dataset (EN), (C) FC features from root_1 dataset (CNN), and (D) FC features from root_2 dataset (CNN). The final number of dimensions selected for the analysis was 2, corresponding to stress values of 0.14, 0.13, 0.01, and 0.01 for EN_root_1, EN_root_2, CNN_root_1, and CNN_root_2 respectively.


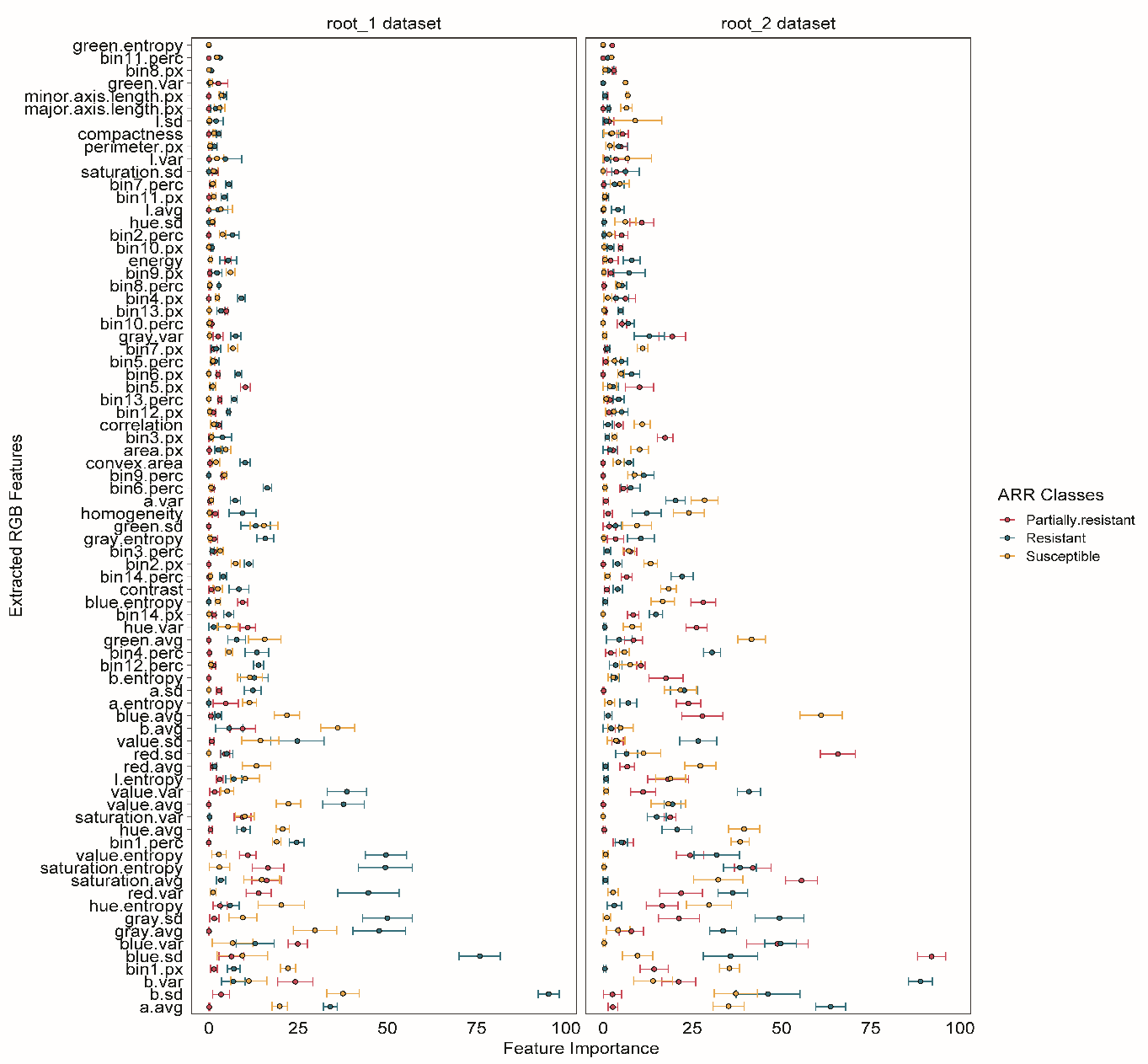


**Figure S2**. Final RGB feature importance evaluated using EN model for root_1 (left panel) and root_2 (right panel).

**Table S1.** Aphanomyces root rot visual disease scoring criteria.

| Visual Disease Scores | Symptoms |
| --- | --- |
| 0.0 | White roots with no visible symptoms |
| 0.5 | Less than 5% of discolored lesions on the entire root |
| 1.0 | 5–15% of discolored lesions on the entire root |
| 1.5 | 15–25% of discolored lesions on the entire root |
| 2.0 | 25–50% minor discoloration on the entire root |
| 2.5 | 50–75% major discoloration on the entire root |
| 3.0 | More than 75% of brown discoloration on the entire root |
| 3.5 | More than 75% of brown discoloration on entire root system with some symptoms on hypocotyl |
| 4.0 | Brown discoloration on entire root system with shriveled and brown hypocotyl |
| 4.5 | Brown discoloration on entire root system with a shriveled, brown, and soft hypocotyl |
| 5.0 | Dead plant |

**Table S2.** List of root features extracted from RGB images.

| **#** | **RGB features** | **Definition** |
| --- | --- | --- |
| **1** | area.px | Total number of white pixels corresponding to root |
| **2** | convex.area | Total number of white pixels corresponding to the convex hull of root |
| **3** | major.axis.length.px | Length of major axis in pixels |
| **4** | minor.axis.length.px | Length of minor axis in pixels |
| **5** | perimeter.px | Length of root boundary in pixels |
| **6** | compactness | Ratio of projected area in pixels by perimeter in pixels |
| **7** | bin1.px | Total number of pixels in the range 0.05 ≤ Hue ≤ 0.10 and 0.00 ≤ Saturation < 0.50 and 0.00 ≤ Value ≤ 1.00 |
| **8** | bin1.perc | Percentage of pixels in the range 0.05 ≤ Hue ≤ 0.10 and 0.00 ≤ Saturation < 0.50 and 0.00 ≤ Value ≤ 1.00 |
| **9** | bin2.px | Total number of pixels in the range 0.05 ≤ Hue ≤ 0.10 and 0.50 ≤ Saturation ≤ 1.00 and 0.00 ≤ Value ≤ 1.00 |
| **10** | bin2.perc | Percentage of pixels in the range 0.05 ≤ Hue ≤ 0.10 and 0.50 ≤ Saturation ≤ 1.00 and 0.00 ≤ Value ≤ 1.00 |
| **11** | bin3.px | Total number of pixels in the range 0.11 ≤ Hue ≤ 0.15 and 0.00 ≤ Saturation < 0.50 and 0.00 ≤ Value ≤ 1.00 |
| **12** | bin3.perc | Percentage of pixels in the range 0.11 ≤ Hue ≤ 0.15 and 0.00 ≤ Saturation < 0.50 and 0.00 ≤ Value ≤ 1.00 |
| **13** | bin4.px | Total number of pixels in the range 0.11 ≤ Hue ≤ 0.15 and 0.50 ≤ Saturation ≤ 1.00 and 0.00 ≤ Value ≤ 1.00 |
| **14** | bin4.perc | Percentage of pixels in the range 0.11 ≤ Hue ≤ 0.15 and 0.50 ≤ Saturation ≤ 1.00 and 0.00 ≤ Value ≤ 1.00 |
| **15** | bin5.px | Total number of pixels in the range 0.151 ≤ Hue ≤ 0.20 and 0.00 ≤ Saturation < 0.50 and 0.00 ≤ Value ≤ 1.00 |
| **16** | bin5.perc | Percentage of pixels in the range 0.151 ≤ Hue ≤ 0.20 and 0.00 ≤ Saturation < 0.50 and 0.00 ≤ Value ≤ 1.00 |
| **17** | bin6.px | Total number of pixels in the range 0.151 ≤ Hue ≤ 0.20 and 0.50 ≤ Saturation ≤ 1.00 and 0.00 ≤ Value ≤ 1.00 |
| **18** | bin6.perc | Percentage of pixels in the range 0.151 ≤ Hue ≤ 0.20 and 0.50 ≤ Saturation ≤ 1.00 and 0.00 ≤ Value ≤ 1.00 |
| **19** | bin7.px | Total number of pixels in the range 0.21 ≤ Hue ≤ 0.25 and 0.00 ≤ Saturation < 0.50 and 0.00 ≤ Value ≤ 1.00 |
| **20** | bin7.perc | Percentage of pixels in the range 0.21 ≤ Hue ≤ 0.25 and 0.00 ≤ Saturation < 0.50 and 0.00 ≤ Value ≤ 1.00 |
| **21** | bin8.px | Total number of pixels in the range 0.21 ≤ Hue ≤ 0.25 and 0.50 ≤ Saturation ≤ 1.00 and 0.00 ≤ Value ≤ 1.00 |
| **22** | bin8.perc | Percentage of pixels in the range 0.21 ≤ Hue ≤ 0.25 and 0.50 ≤ Saturation ≤ 1.00 and 0.00 ≤ Value ≤ 1.00 |
| **23** | bin9.px | Total number of pixels in the range 0.251 ≤ Hue ≤ 0.30 and 0.00 ≤ Saturation < 0.50 and 0.00 ≤ Value ≤ 1.00 |
| **24** | bin9.perc | Percentage of pixels in the range 0.251 ≤ Hue ≤ 0.30 and 0.00 ≤ Saturation < 0.50 and 0.00 ≤ Value ≤ 1.00 |
| **25** | bin10.px | Total number of pixels in the range 0.251 ≤ Hue ≤ 0.30 and 0.50 ≤ Saturation ≤ 1.00 and 0.00 ≤ Value ≤ 1.00 |
| **26** | bin10.perc | Percentage of pixels in the range 0.251 ≤ Hue ≤ 0.30 and 0.50 ≤ Saturation ≤ 1.00 and 0.00 ≤ Value ≤ 1.00 |
| **27** | bin11.px | Total number of pixels in the range 0.31 ≤ Hue ≤ 0.35 and 0.00 ≤ Saturation < 0.50 and 0.00 ≤ Value ≤ 1.00 |
| **28** | bin11.perc | Percentage of pixels in the range 0.31 ≤ Hue ≤ 0.35 and 0.00 ≤ Saturation < 0.50 and 0.00 ≤ Value ≤ 1.00 |
| **29** | bin12.px | Total number of pixels in the range 0.31 ≤ Hue ≤ 0.35 and 0.50 ≤ Saturation ≤ 1.00 and 0.00 ≤ Value ≤ 1.00 |
| **30** | bin12.px | Total number of pixels in the range 0.31 ≤ Hue ≤ 0.35 and 0.50 ≤ Saturation ≤ 1.00 and 0.00 ≤ Value ≤ 1.00 |
| **31** | bin13.px | Total number of pixels in the range 0.351 ≤ Hue ≤ 0.40 and 0.00 ≤ Saturation < 0.50 and 0.00 ≤ Value ≤ 1.00 |
| **32** | bin13.perc | Percentage of pixels in the range 0.351 ≤ Hue ≤ 0.40 and 0.00 ≤ Saturation < 0.50 and 0.00 ≤ Value ≤ 1.00 |
| **33** | bin14.px | Total number of pixels in the range 0.351 ≤ Hue ≤ 0.40 and 0.50 ≤ Saturation ≤ 1.00 and 0.00 ≤ Value ≤ 1.00 |
| **34** | bin14.perc | Percentage of pixels in the range 0.351 ≤ Hue ≤ 0.40 and 0.50 ≤ Saturation ≤ 1.00 and 0.00 ≤ Value ≤ 1.00 |
| **35** | hue.avg | Average intensity in Hue channel (RGB converted to HSV) |
| **36** | hue.sd | Standard deviation of intensity in Hue channel (RGB converted to HSV) |
| **37** | hue.var | Variance of intensity in Hue channel (RGB converted to HSV) |
| **38** | hue.entropy | Entropy of intensity in Hue channel (RGB converted to HSV) |
| **39** | saturation.avg | Average intensity in Saturation channel (RGB converted to HSV) |
| **40** | saturation.sd | Standard deviation of intensity in Saturation channel (RGB converted to HSV) |
| **41** | saturation.var | Variance of intensity in Saturation channel (RGB converted to HSV) |
| **42** | saturation.entropy | Entropy of intensity in Saturation channel (RGB converted to HSV) |
| **43** | value.avg | Average intensity in Value channel (RGB converted to HSV) |
| **44** | value.sd | Standard deviation of intensity in Value channel (RGB converted to HSV) |
| **45** | value.var | Variance of intensity in Value channel (RGB converted to HSV) |
| **46** | value.entropy | Entropy of intensity in Value channel (RGB converted to HSV) |
| **47** | red.avg | Average intensity in Red channel (RGB) |
| **48** | red.sd | Standard deviation of intensity in Red channel (RGB) |
| **49** | red.var | Variance of intensity in Red channel (RGB) |
| **50** | red.entropy | Entropy of intensity in Red channel (RGB) |
| **51** | green.avg | Average intensity in Green channel (RGB) |
| **52** | green.sd | Standard deviation of intensity in Green channel (RGB) |
| **53** | green.var | Variance of intensity in Green channel (RGB) |
| **54** | green.entropy | Entropy of intensity in Green channel (RGB) |
| **55** | blue.avg | Average intensity in Blue channel (RGB) |
| **56** | blue.sd | Standard deviation of intensity in Blue channel (RGB) |
| **57** | blue.var | Variance of intensity in Blue channel (RGB) |
| **58** | blue.entropy | Entropy of intensity in Blue channel (RGB) |
| **59** | gray.avg | Average intensity in grayscale image (RGB converted to grayscale) |
| **60** | gray.sd | Standard deviation of intensity in grayscale image (RGB converted to grayscale) |
| **61** | gray.var | Variance of intensity in grayscale image (RGB converted to grayscale) |
| **62** | gray.entropy | Entropy of intensity in grayscale image (RGB converted to grayscale) |
| **63** | l.avg | Average of intensity in L channel (RGB converted to Lab)× |
| **64** | l.sd | Standard deviation of intensity in L channel (RGB converted to Lab) |
| **65** | l.var | Variance of intensity in L channel (RGB converted to Lab) |
| **66** | l.entropy | Entropy of intensity in L channel (RGB converted to Lab) |
| **67** | a.avg | Average of intensity in a channel (RGB converted to Lab) |
| **68** | a.sd | Standard deviation of intensity in a channel (RGB converted to Lab) |
| **69** | a.var | Variance of intensity in a channel (RGB converted to Lab) |
| **70** | a.entropy | Entropy of intensity in a channel (RGB converted to Lab) |
| **71** | b.avg | Average of intensity in b channel (RGB converted to Lab) |
| **72** | b.sd | Standard deviation of intensity in b channel (RGB converted to Lab) |
| **73** | b.var | Variance of intensity in b channel (RGB converted to Lab) |
| **74** | b.entropy | Entropy of intensity in b channel (RGB converted to Lab) |
| **75** | contrast | Local variations in the gray-level-co-occurrence matrix* |
| **76** | homogeneity | Closeness of the distribution of elements in the gray-level-co-occurrence matrix to the gray-level-co-occurrence matrix diagonal* |
| **77** | correlation | Joint probability occurrence of the specified pixel pairs* |
| **78** | energy | Sum of squared elements or uniformity in the gray-level-co-occurrence matrix* |
|  |  |  |

* MATLAB https://www.mathworks.com/help/images/texture-analysis-1.html (Accessed on November 18, 2019)

**Table S3.** Number of selected features based on their importance scores.

| **Dataset** | **Random run number** | **Number of features** | **Validation accuracy** |
| --- | --- | --- | --- |
| root_1 | 1 | 35 | 0.79 |
| root_1 | 2 | 55 | 0.78 |
| root_1 | 3 | 78 | 0.76 |
| root_1 | 4 | 70 | 0.78 |
| root_1 | 5 | 15 | 0.76 |
| root_1 | 6 | 25 | 0.79 |
| root_1 | 7 | 25 | 0.76 |
| root_1 | 8 | 60 | 0.82 |
| root_1 | 9 | 25 | 0.80 |
| root_1 | 10 | 65 | 0.77 |
| root_2 | 1 | 70 | 0.92 |
| root_2 | 2 | 35 | 0.94 |
| root_2 | 3 | 50 | 0.90 |
| root_2 | 4 | 65 | 0.94 |
| root_2 | 5 | 35 | 0.92 |
| root_2 | 6 | 50 | 0.92 |
| root_2 | 7 | 70 | 0.91 |
| root_2 | 8 | 60 | 0.93 |
| root_2 | 9 | 78 | 0.92 |
| root_2 | 10 | 60 | 0.92 |

**Table S4**. CNN performance during training and validation (averaged across the 10 random runs).

| **Dataset** | **Epoch** | **Iteration** | **MiniBatchAcc** | **ValidationAcc** | **MiniBatchLoss** | **ValidationLoss** | **BaseLearningRate** |
| --- | --- | --- | --- | --- | --- | --- | --- |
| root_1 | 1 | 1 | 0.28 | 0.38 | 1.91 | 1.62 | 1.00E-04 |
| root_1 | 1 | 20 | 0.38 | 0.44 | 2.56 | 2.32 | 1.00E-04 |
| root_1 | 1 | 40 | 0.38 | 0.43 | 3.00 | 2.74 | 1.00E-04 |
| root_1 | 1 | 50 | 0.47 | NA | 2.32 | NA | 1.00E-04 |
| root_1 | 1 | 60 | 0.35 | 0.45 | 2.69 | 2.22 | 1.00E-04 |
| root_1 | 1 | 80 | 0.50 | 0.48 | 2.01 | 2.13 | 1.00E-04 |
| root_1 | 1 | 100 | 0.49 | 0.49 | 2.00 | 1.76 | 1.00E-04 |
| root_1 | 1 | 120 | 0.47 | 0.49 | 1.71 | 1.59 | 1.00E-04 |
| root_1 | 1 | 140 | 0.48 | 0.48 | 1.92 | 1.92 | 1.00E-04 |
| root_1 | 1 | 150 | 0.47 | NA | 1.65 | NA | 1.00E-04 |
| root_1 | 1 | 160 | 0.52 | 0.51 | 1.49 | 1.75 | 1.00E-04 |
| root_1 | 2 | 180 | 0.52 | 0.53 | 1.64 | 1.46 | 1.00E-04 |
| root_1 | 2 | 200 | 0.53 | 0.53 | 1.48 | 1.59 | 1.00E-04 |
| root_1 | 2 | 220 | 0.56 | 0.55 | 1.40 | 1.43 | 1.00E-04 |
| root_1 | 2 | 240 | 0.53 | 0.54 | 1.37 | 1.51 | 1.00E-04 |
| root_1 | 2 | 250 | 0.51 | NA | 1.78 | NA | 1.00E-04 |
| root_1 | 2 | 260 | 0.54 | 0.57 | 1.46 | 1.37 | 1.00E-04 |
| root_1 | 2 | 280 | 0.55 | 0.56 | 1.28 | 1.29 | 1.00E-04 |
| root_1 | 2 | 300 | 0.58 | 0.58 | 1.44 | 1.43 | 1.00E-04 |
| root_1 | 2 | 320 | 0.51 | 0.56 | 1.43 | 1.31 | 1.00E-04 |
| root_1 | 3 | 340 | 0.53 | 0.58 | 1.50 | 1.33 | 1.00E-04 |
| root_1 | 3 | 350 | 0.57 | NA | 1.10 | NA | 1.00E-04 |
| root_1 | 3 | 360 | 0.55 | 0.56 | 1.32 | 1.41 | 1.00E-04 |
| root_1 | 3 | 380 | 0.51 | 0.59 | 1.39 | 1.24 | 1.00E-04 |
| root_1 | 3 | 400 | 0.60 | 0.60 | 1.18 | 1.24 | 1.00E-04 |
| root_1 | 3 | 420 | 0.61 | 0.58 | 1.29 | 1.20 | 1.00E-04 |
| root_1 | 3 | 440 | 0.58 | 0.58 | 1.43 | 1.33 | 1.00E-04 |
| root_1 | 3 | 450 | 0.58 | NA | 1.37 | NA | 1.00E-04 |
| root_1 | 3 | 460 | 0.63 | 0.57 | 1.13 | 1.31 | 1.00E-04 |
| root_1 | 3 | 480 | 0.58 | 0.61 | 1.39 | 1.29 | 1.00E-04 |
| root_1 | 4 | 500 | 0.56 | 0.58 | 1.47 | 1.27 | 1.00E-04 |
| root_1 | 4 | 520 | 0.56 | 0.61 | 1.39 | 1.18 | 1.00E-04 |
| root_1 | 4 | 540 | 0.58 | 0.61 | 1.32 | 1.10 | 1.00E-04 |
| root_1 | 4 | 550 | 0.60 | NA | 1.34 | NA | 1.00E-04 |
| root_1 | 4 | 560 | 0.62 | 0.61 | 1.34 | 1.17 | 1.00E-04 |
| root_1 | 4 | 580 | 0.53 | 0.58 | 1.37 | 1.22 | 1.00E-04 |
| root_1 | 4 | 600 | 0.55 | 0.61 | 1.34 | 1.13 | 1.00E-04 |
| root_1 | 4 | 620 | 0.62 | 0.63 | 1.15 | 1.13 | 1.00E-04 |
| root_1 | 4 | 640 | 0.60 | 0.62 | 1.15 | 1.10 | 1.00E-04 |
| root_1 | 5 | 650 | 0.56 | NA | 1.28 | NA | 1.00E-04 |
| root_1 | 5 | 660 | 0.60 | 0.62 | 1.08 | 1.02 | 1.00E-04 |
| root_1 | 5 | 680 | 0.55 | 0.60 | 1.27 | 1.21 | 1.00E-04 |
| root_1 | 5 | 700 | 0.56 | 0.61 | 1.28 | 1.11 | 1.00E-04 |
| root_1 | 5 | 720 | 0.55 | 0.59 | 1.28 | 1.19 | 1.00E-04 |
| root_1 | 5 | 740 | 0.64 | 0.62 | 0.94 | 1.11 | 1.00E-04 |
| root_1 | 5 | 750 | 0.62 | NA | 1.00 | NA | 1.00E-04 |
| root_1 | 5 | 760 | 0.58 | 0.64 | 1.08 | 0.99 | 1.00E-04 |
| root_1 | 5 | 780 | 0.58 | 0.61 | 1.21 | 1.13 | 1.00E-04 |
| root_1 | 5 | 800 | 0.53 | 0.62 | 1.60 | 1.12 | 1.00E-04 |
| root_1 | 6 | 820 | 0.62 | 0.63 | 1.03 | 1.05 | 1.00E-04 |
| root_1 | 6 | 840 | 0.64 | 0.62 | 1.05 | 1.05 | 1.00E-04 |
| root_1 | 6 | 850 | 0.55 | NA | 1.45 | NA | 1.00E-04 |
| root_1 | 6 | 860 | 0.59 | 0.63 | 1.08 | 1.05 | 1.00E-04 |
| root_1 | 6 | 880 | 0.61 | 0.61 | 1.04 | 1.12 | 1.00E-04 |
| root_1 | 6 | 900 | 0.60 | 0.64 | 1.28 | 0.98 | 1.00E-04 |
| root_1 | 6 | 920 | 0.57 | 0.63 | 1.25 | 1.02 | 1.00E-04 |
| root_1 | 6 | 940 | 0.61 | 0.62 | 1.15 | 1.14 | 1.00E-04 |
| root_1 | 6 | 950 | 0.56 | NA | 1.25 | NA | 1.00E-04 |
| root_1 | 6 | 960 | 0.56 | 0.64 | 1.17 | 1.07 | 1.00E-04 |
| root_1 | 7 | 980 | 0.63 | 0.66 | 1.03 | 0.93 | 1.00E-04 |
| root_1 | 7 | 1000 | 0.59 | 0.64 | 1.12 | 0.98 | 1.00E-04 |
| root_1 | 7 | 1020 | 0.63 | 0.61 | 1.11 | 1.06 | 1.00E-04 |
| root_1 | 7 | 1040 | 0.64 | 0.64 | 1.10 | 1.03 | 1.00E-04 |
| root_1 | 7 | 1050 | 0.55 | NA | 1.13 | NA | 1.00E-04 |
| root_1 | 7 | 1060 | 0.64 | 0.65 | 0.97 | 0.94 | 1.00E-04 |
| root_1 | 7 | 1080 | 0.62 | 0.64 | 1.10 | 0.95 | 1.00E-04 |
| root_1 | 7 | 1100 | 0.62 | 0.64 | 0.94 | 0.99 | 1.00E-04 |
| root_1 | 7 | 1120 | 0.59 | 0.64 | 1.22 | 0.99 | 1.00E-04 |
| root_1 | 8 | 1140 | 0.55 | 0.63 | 1.31 | 1.04 | 1.00E-04 |
| root_1 | 8 | 1150 | 0.60 | NA | 0.99 | NA | 1.00E-04 |
| root_1 | 8 | 1160 | 0.54 | 0.63 | 1.23 | 1.03 | 1.00E-04 |
| root_1 | 8 | 1180 | 0.57 | 0.64 | 1.16 | 1.01 | 1.00E-04 |
| root_1 | 8 | 1200 | 0.59 | 0.63 | 1.17 | 1.00 | 1.00E-04 |
| root_1 | 8 | 1220 | 0.61 | 0.64 | 1.12 | 0.97 | 1.00E-04 |
| root_1 | 8 | 1240 | 0.59 | 0.64 | 1.34 | 1.05 | 1.00E-04 |
| root_1 | 8 | 1250 | 0.63 | NA | 1.02 | NA | 1.00E-04 |
| root_1 | 8 | 1260 | 0.64 | 0.64 | 1.07 | 1.05 | 1.00E-04 |
| root_1 | 8 | 1280 | 0.60 | 0.63 | 1.15 | 1.05 | 1.00E-04 |
| root_1 | 9 | 1300 | 0.61 | 0.60 | 1.15 | 1.21 | 1.00E-04 |
| root_1 | 9 | 1320 | 0.59 | 0.64 | 1.06 | 0.97 | 1.00E-04 |
| root_1 | 9 | 1340 | 0.54 | 0.64 | 1.26 | 1.03 | 1.00E-04 |
| root_1 | 9 | 1350 | 0.65 | NA | 1.00 | NA | 1.00E-04 |
| root_1 | 9 | 1360 | 0.65 | 0.64 | 0.98 | 0.96 | 1.00E-04 |
| root_1 | 9 | 1380 | 0.59 | 0.63 | 1.10 | 1.03 | 1.00E-04 |
| root_1 | 9 | 1400 | 0.65 | 0.63 | 1.07 | 1.02 | 1.00E-04 |
| root_1 | 9 | 1420 | 0.58 | 0.61 | 1.07 | 1.00 | 1.00E-04 |
| root_1 | 9 | 1440 | 0.64 | 0.64 | 0.97 | 0.99 | 1.00E-04 |
| root_1 | 10 | 1450 | 0.63 | NA | 0.97 | NA | 1.00E-04 |
| root_1 | 10 | 1460 | 0.66 | 0.63 | 0.90 | 1.00 | 1.00E-04 |
| root_1 | 10 | 1480 | 0.59 | 0.63 | 1.05 | 0.99 | 1.00E-04 |
| root_1 | 10 | 1500 | 0.59 | 0.64 | 1.14 | 1.04 | 1.00E-04 |
| root_1 | 10 | 1520 | 0.56 | 0.64 | 1.21 | 0.99 | 1.00E-04 |
| root_1 | 10 | 1540 | 0.63 | 0.64 | 0.91 | 1.00 | 1.00E-04 |
| root_1 | 10 | 1550 | 0.60 | NA | 1.10 | NA | 1.00E-04 |
| root_1 | 10 | 1560 | 0.60 | 0.64 | 1.08 | 0.95 | 1.00E-04 |
| root_1 | 10 | 1580 | 0.63 | 0.65 | 1.04 | 0.94 | 1.00E-04 |
| root_1 | 10 | 1600 | 0.64 | 0.64 | 1.08 | 0.96 | 1.00E-04 |
| root_1 | 11 | 1620 | 0.55 | 0.57 | 1.92 | 1.65 | 1.00E-05 |
| root_1 | 11 | 1640 | 0.67 | 0.65 | 1.10 | 0.94 | 1.00E-05 |
| root_1 | 11 | 1650 | 0.62 | NA | 0.97 | NA | 1.00E-05 |
| root_1 | 11 | 1660 | 0.62 | 0.68 | 0.99 | 0.81 | 1.00E-05 |
| root_1 | 11 | 1680 | 0.65 | 0.68 | 0.86 | 0.79 | 1.00E-05 |
| root_1 | 11 | 1700 | 0.66 | 0.69 | 0.83 | 0.77 | 1.00E-05 |
| root_1 | 11 | 1720 | 0.68 | 0.69 | 0.77 | 0.75 | 1.00E-05 |
| root_1 | 11 | 1740 | 0.61 | 0.69 | 0.87 | 0.75 | 1.00E-05 |
| root_1 | 11 | 1750 | 0.67 | NA | 0.79 | NA | 1.00E-05 |
| root_1 | 11 | 1760 | 0.69 | 0.69 | 0.69 | 0.75 | 1.00E-05 |
| root_1 | 12 | 1780 | 0.64 | 0.69 | 0.84 | 0.75 | 1.00E-05 |
| root_1 | 12 | 1800 | 0.62 | 0.69 | 0.91 | 0.72 | 1.00E-05 |
| root_1 | 12 | 1820 | 0.68 | 0.70 | 0.83 | 0.72 | 1.00E-05 |
| root_1 | 12 | 1840 | 0.68 | 0.70 | 0.70 | 0.73 | 1.00E-05 |
| root_1 | 12 | 1850 | 0.65 | NA | 0.80 | NA | 1.00E-05 |
| root_1 | 12 | 1860 | 0.64 | 0.69 | 0.81 | 0.73 | 1.00E-05 |
| root_1 | 12 | 1880 | 0.68 | 0.70 | 0.83 | 0.71 | 1.00E-05 |
| root_1 | 12 | 1900 | 0.63 | 0.70 | 0.77 | 0.71 | 1.00E-05 |
| root_1 | 12 | 1920 | 0.66 | 0.69 | 0.86 | 0.71 | 1.00E-05 |
| root_1 | 13 | 1940 | 0.68 | 0.69 | 0.68 | 0.72 | 1.00E-05 |
| root_1 | 13 | 1950 | 0.71 | NA | 0.67 | NA | 1.00E-05 |
| root_1 | 13 | 1960 | 0.69 | 0.70 | 0.72 | 0.70 | 1.00E-05 |
| root_1 | 13 | 1980 | 0.69 | 0.70 | 0.68 | 0.70 | 1.00E-05 |
| root_1 | 13 | 2000 | 0.58 | 0.69 | 0.87 | 0.72 | 1.00E-05 |
| root_1 | 13 | 2020 | 0.60 | 0.70 | 0.92 | 0.71 | 1.00E-05 |
| root_1 | 13 | 2040 | 0.68 | 0.70 | 0.80 | 0.69 | 1.00E-05 |
| root_1 | 13 | 2050 | 0.65 | NA | 0.70 | NA | 1.00E-05 |
| root_1 | 13 | 2060 | 0.61 | 0.70 | 0.96 | 0.70 | 1.00E-05 |
| root_1 | 13 | 2080 | 0.67 | 0.69 | 0.79 | 0.70 | 1.00E-05 |
| root_1 | 14 | 2100 | 0.68 | 0.70 | 0.80 | 0.69 | 1.00E-05 |
| root_1 | 14 | 2120 | 0.69 | 0.70 | 0.67 | 0.68 | 1.00E-05 |
| root_1 | 14 | 2140 | 0.68 | 0.70 | 0.68 | 0.69 | 1.00E-05 |
| root_1 | 14 | 2150 | 0.61 | NA | 0.87 | NA | 1.00E-05 |
| root_1 | 14 | 2160 | 0.65 | 0.70 | 0.71 | 0.68 | 1.00E-05 |
| root_1 | 14 | 2180 | 0.66 | 0.69 | 0.77 | 0.70 | 1.00E-05 |
| root_1 | 14 | 2200 | 0.68 | 0.70 | 0.75 | 0.69 | 1.00E-05 |
| root_1 | 14 | 2220 | 0.63 | 0.70 | 0.83 | 0.68 | 1.00E-05 |
| root_1 | 14 | 2240 | 0.75 | 0.70 | 0.63 | 0.69 | 1.00E-05 |
| root_1 | 14 | 2250 | 0.66 | NA | 0.74 | NA | 1.00E-05 |
| root_1 | 15 | 2260 | 0.69 | 0.69 | 0.71 | 0.70 | 1.00E-05 |
| root_1 | 15 | 2280 | 0.64 | 0.70 | 0.80 | 0.69 | 1.00E-05 |
| root_1 | 15 | 2300 | 0.67 | 0.70 | 0.69 | 0.68 | 1.00E-05 |
| root_1 | 15 | 2320 | 0.69 | 0.70 | 0.74 | 0.68 | 1.00E-05 |
| root_1 | 15 | 2340 | 0.63 | 0.69 | 0.82 | 0.68 | 1.00E-05 |
| root_1 | 15 | 2350 | 0.67 | NA | 0.80 | NA | 1.00E-05 |
| root_1 | 15 | 2360 | 0.63 | 0.70 | 0.71 | 0.69 | 1.00E-05 |
| root_1 | 15 | 2380 | 0.70 | 0.70 | 0.64 | 0.68 | 1.00E-05 |
| root_1 | 15 | 2400 | 0.64 | 0.70 | 0.77 | 0.69 | 1.00E-05 |
| root_1 | 16 | 2420 | 0.65 | 0.70 | 0.73 | 0.68 | 1.00E-05 |
| root_1 | 16 | 2440 | 0.65 | 0.70 | 0.72 | 0.68 | 1.00E-05 |
| root_1 | 16 | 2450 | 0.67 | NA | 0.69 | NA | 1.00E-05 |
| root_1 | 16 | 2460 | 0.64 | 0.70 | 0.93 | 0.67 | 1.00E-05 |
| root_1 | 16 | 2480 | 0.68 | 0.70 | 0.69 | 0.68 | 1.00E-05 |
| root_1 | 16 | 2500 | 0.66 | 0.70 | 0.78 | 0.67 | 1.00E-05 |
| root_1 | 16 | 2520 | 0.65 | 0.70 | 0.76 | 0.68 | 1.00E-05 |
| root_1 | 16 | 2540 | 0.65 | 0.70 | 0.74 | 0.67 | 1.00E-05 |
| root_1 | 16 | 2550 | 0.69 | NA | 0.71 | NA | 1.00E-05 |
| root_1 | 16 | 2560 | 0.69 | 0.70 | 0.64 | 0.67 | 1.00E-05 |
| root_1 | 17 | 2580 | 0.69 | 0.70 | 0.69 | 0.67 | 1.00E-05 |
| root_1 | 17 | 2600 | 0.70 | 0.69 | 0.70 | 0.68 | 1.00E-05 |
| root_1 | 17 | 2620 | 0.65 | 0.70 | 0.73 | 0.67 | 1.00E-05 |
| root_1 | 17 | 2640 | 0.69 | 0.70 | 0.70 | 0.67 | 1.00E-05 |
| root_1 | 17 | 2650 | 0.67 | NA | 0.75 | NA | 1.00E-05 |
| root_1 | 17 | 2660 | 0.63 | 0.70 | 0.75 | 0.68 | 1.00E-05 |
| root_1 | 17 | 2680 | 0.68 | 0.70 | 0.67 | 0.67 | 1.00E-05 |
| root_1 | 17 | 2700 | 0.62 | 0.70 | 0.80 | 0.68 | 1.00E-05 |
| root_1 | 17 | 2720 | 0.65 | 0.70 | 0.72 | 0.67 | 1.00E-05 |
| root_1 | 18 | 2740 | 0.66 | 0.70 | 0.78 | 0.68 | 1.00E-05 |
| root_1 | 18 | 2750 | 0.71 | NA | 0.76 | NA | 1.00E-05 |
| root_1 | 18 | 2760 | 0.66 | 0.70 | 0.78 | 0.66 | 1.00E-05 |
| root_1 | 18 | 2780 | 0.65 | 0.70 | 0.75 | 0.68 | 1.00E-05 |
| root_1 | 18 | 2800 | 0.68 | 0.70 | 0.67 | 0.67 | 1.00E-05 |
| root_1 | 18 | 2820 | 0.74 | 0.70 | 0.61 | 0.66 | 1.00E-05 |
| root_1 | 18 | 2840 | 0.74 | 0.70 | 0.64 | 0.66 | 1.00E-05 |
| root_1 | 18 | 2850 | 0.66 | NA | 0.74 | NA | 1.00E-05 |
| root_1 | 18 | 2860 | 0.64 | 0.70 | 0.76 | 0.67 | 1.00E-05 |
| root_1 | 18 | 2880 | 0.67 | 0.70 | 0.71 | 0.66 | 1.00E-05 |
| root_1 | 19 | 2900 | 0.71 | 0.71 | 0.79 | 0.66 | 1.00E-05 |
| root_1 | 19 | 2920 | 0.65 | 0.71 | 0.74 | 0.67 | 1.00E-05 |
| root_1 | 19 | 2940 | 0.70 | 0.70 | 0.68 | 0.66 | 1.00E-05 |
| root_1 | 19 | 2950 | 0.65 | NA | 0.73 | NA | 1.00E-05 |
| root_1 | 19 | 2960 | 0.63 | 0.71 | 0.84 | 0.66 | 1.00E-05 |
| root_1 | 19 | 2980 | 0.69 | 0.71 | 0.69 | 0.66 | 1.00E-05 |
| root_1 | 19 | 3000 | 0.70 | 0.70 | 0.69 | 0.66 | 1.00E-05 |
| root_1 | 19 | 3020 | 0.68 | 0.70 | 0.76 | 0.67 | 1.00E-05 |
| root_1 | 19 | 3040 | 0.69 | 0.70 | 0.65 | 0.66 | 1.00E-05 |
| root_1 | 19 | 3050 | 0.71 | NA | 0.59 | NA | 1.00E-05 |
| root_1 | 20 | 3060 | 0.67 | 0.70 | 0.78 | 0.67 | 1.00E-05 |
| root_1 | 20 | 3080 | 0.69 | 0.70 | 0.73 | 0.67 | 1.00E-05 |
| root_1 | 20 | 3100 | 0.68 | 0.70 | 0.72 | 0.67 | 1.00E-05 |
| root_1 | 20 | 3120 | 0.67 | 0.70 | 0.76 | 0.66 | 1.00E-05 |
| root_1 | 20 | 3140 | 0.69 | 0.70 | 0.71 | 0.66 | 1.00E-05 |
| root_1 | 20 | 3150 | 0.68 | NA | 0.84 | NA | 1.00E-05 |
| root_1 | 20 | 3160 | 0.71 | 0.70 | 0.67 | 0.66 | 1.00E-05 |
| root_1 | 20 | 3180 | 0.72 | 0.71 | 0.66 | 0.65 | 1.00E-05 |
| root_1 | 20 | 3200 | 0.72 | 0.71 | 0.65 | 0.65 | 1.00E-05 |
| root_1 | 20 | 3220 | 0.67 | 0.71 | 0.76 | 0.67 | 1.00E-05 |
| root_1 | 21 | 3240 | 0.69 | 0.70 | 0.77 | 0.68 | 1.00E-06 |
| root_1 | 21 | 3250 | 0.71 | NA | 0.61 | NA | 1.00E-06 |
| root_1 | 21 | 3260 | 0.68 | 0.71 | 0.72 | 0.65 | 1.00E-06 |
| root_1 | 21 | 3280 | 0.71 | 0.71 | 0.66 | 0.65 | 1.00E-06 |
| root_1 | 21 | 3300 | 0.73 | 0.71 | 0.60 | 0.65 | 1.00E-06 |
| root_1 | 21 | 3320 | 0.69 | 0.71 | 0.74 | 0.65 | 1.00E-06 |
| root_1 | 21 | 3340 | 0.61 | 0.71 | 0.84 | 0.65 | 1.00E-06 |
| root_1 | 21 | 3350 | 0.70 | NA | 0.67 | NA | 1.00E-06 |
| root_1 | 21 | 3360 | 0.61 | 0.71 | 0.77 | 0.65 | 1.00E-06 |
| root_1 | 21 | 3380 | 0.70 | 0.72 | 0.62 | 0.65 | 1.00E-06 |
| root_1 | 22 | 3400 | 0.70 | 0.71 | 0.63 | 0.65 | 1.00E-06 |
| root_1 | 22 | 3420 | 0.66 | 0.71 | 0.68 | 0.64 | 1.00E-06 |
| root_1 | 22 | 3440 | 0.67 | 0.71 | 0.66 | 0.65 | 1.00E-06 |
| root_1 | 22 | 3450 | 0.71 | NA | 0.69 | NA | 1.00E-06 |
| root_1 | 22 | 3460 | 0.68 | 0.71 | 0.71 | 0.65 | 1.00E-06 |
| root_1 | 22 | 3480 | 0.64 | 0.72 | 0.70 | 0.65 | 1.00E-06 |
| root_1 | 22 | 3500 | 0.71 | 0.71 | 0.61 | 0.64 | 1.00E-06 |
| root_1 | 22 | 3520 | 0.67 | 0.72 | 0.73 | 0.64 | 1.00E-06 |
| root_1 | 22 | 3540 | 0.73 | 0.71 | 0.65 | 0.64 | 1.00E-06 |
| root_1 | 23 | 3550 | 0.69 | NA | 0.71 | NA | 1.00E-06 |
| root_1 | 23 | 3560 | 0.67 | 0.71 | 0.67 | 0.64 | 1.00E-06 |
| root_1 | 23 | 3580 | 0.62 | 0.71 | 0.85 | 0.65 | 1.00E-06 |
| root_1 | 23 | 3600 | 0.66 | 0.71 | 0.72 | 0.65 | 1.00E-06 |
| root_1 | 23 | 3620 | 0.68 | 0.71 | 0.70 | 0.65 | 1.00E-06 |
| root_1 | 23 | 3640 | 0.69 | 0.71 | 0.71 | 0.65 | 1.00E-06 |
| root_1 | 23 | 3650 | 0.67 | NA | 0.69 | NA | 1.00E-06 |
| root_1 | 23 | 3660 | 0.65 | 0.71 | 0.74 | 0.64 | 1.00E-06 |
| root_1 | 23 | 3680 | 0.70 | 0.71 | 0.65 | 0.64 | 1.00E-06 |
| root_1 | 23 | 3700 | 0.69 | 0.71 | 0.68 | 0.64 | 1.00E-06 |
| root_1 | 24 | 3720 | 0.64 | 0.72 | 0.73 | 0.64 | 1.00E-06 |
| root_1 | 24 | 3740 | 0.71 | 0.72 | 0.64 | 0.64 | 1.00E-06 |
| root_1 | 24 | 3750 | 0.69 | NA | 0.65 | NA | 1.00E-06 |
| root_1 | 24 | 3760 | 0.65 | 0.72 | 0.66 | 0.64 | 1.00E-06 |
| root_1 | 24 | 3780 | 0.67 | 0.71 | 0.67 | 0.64 | 1.00E-06 |
| root_1 | 24 | 3800 | 0.71 | 0.72 | 0.69 | 0.65 | 1.00E-06 |
| root_1 | 24 | 3820 | 0.71 | 0.72 | 0.70 | 0.64 | 1.00E-06 |
| root_1 | 24 | 3840 | 0.63 | 0.72 | 0.77 | 0.65 | 1.00E-06 |
| root_1 | 24 | 3850 | 0.67 | NA | 0.83 | NA | 1.00E-06 |
| root_1 | 24 | 3860 | 0.66 | 0.72 | 0.74 | 0.64 | 1.00E-06 |
| root_1 | 25 | 3880 | 0.63 | 0.72 | 0.83 | 0.65 | 1.00E-06 |
| root_1 | 25 | 3900 | 0.64 | 0.71 | 0.77 | 0.64 | 1.00E-06 |
| root_1 | 25 | 3920 | 0.61 | 0.72 | 0.75 | 0.64 | 1.00E-06 |
| root_1 | 25 | 3940 | 0.68 | 0.72 | 0.64 | 0.64 | 1.00E-06 |
| root_1 | 25 | 3950 | 0.68 | NA | 0.61 | NA | 1.00E-06 |
| root_1 | 25 | 3960 | 0.68 | 0.73 | 0.69 | 0.64 | 1.00E-06 |
| root_1 | 25 | 3980 | 0.67 | 0.71 | 0.79 | 0.65 | 1.00E-06 |
| root_1 | 25 | 4000 | 0.76 | 0.72 | 0.55 | 0.65 | 1.00E-06 |
| root_1 | 25 | 4020 | 0.77 | 0.72 | 0.55 | 0.65 | 1.00E-06 |
| root_1 | 26 | 4040 | 0.72 | 0.72 | 0.73 | 0.65 | 1.00E-06 |
| root_1 | 26 | 4050 | 0.68 | NA | 0.73 | NA | 1.00E-06 |
| root_1 | 26 | 4060 | 0.61 | 0.73 | 0.96 | 0.64 | 1.00E-06 |
| root_1 | 26 | 4080 | 0.64 | 0.72 | 0.84 | 0.64 | 1.00E-06 |
| root_1 | 26 | 4100 | 0.73 | 0.72 | 0.66 | 0.64 | 1.00E-06 |
| root_1 | 26 | 4120 | 0.68 | 0.72 | 0.68 | 0.64 | 1.00E-06 |
| root_1 | 26 | 4140 | 0.72 | 0.72 | 0.63 | 0.64 | 1.00E-06 |
| root_1 | 26 | 4150 | 0.65 | NA | 0.69 | NA | 1.00E-06 |
| root_1 | 26 | 4160 | 0.74 | 0.73 | 0.63 | 0.65 | 1.00E-06 |
| root_1 | 26 | 4180 | 0.70 | 0.72 | 0.68 | 0.65 | 1.00E-06 |
| root_1 | 27 | 4200 | 0.68 | 0.72 | 0.69 | 0.66 | 1.00E-06 |
| root_1 | 27 | 4220 | 0.68 | 0.71 | 0.77 | 0.65 | 1.00E-06 |
| root_1 | 27 | 4240 | 0.70 | 0.73 | 0.71 | 0.65 | 1.00E-06 |
| root_1 | 27 | 4250 | 0.66 | NA | 0.74 | NA | 1.00E-06 |
| root_1 | 27 | 4260 | 0.61 | 0.72 | 0.90 | 0.64 | 1.00E-06 |
| root_1 | 27 | 4280 | 0.74 | 0.72 | 0.62 | 0.64 | 1.00E-06 |
| root_1 | 27 | 4300 | 0.60 | 0.72 | 0.82 | 0.65 | 1.00E-06 |
| root_1 | 27 | 4320 | 0.68 | 0.72 | 0.75 | 0.64 | 1.00E-06 |
| root_1 | 27 | 4340 | 0.64 | 0.72 | 0.79 | 0.64 | 1.00E-06 |
| root_1 | 28 | 4350 | 0.70 | NA | 0.66 | NA | 1.00E-06 |
| root_1 | 28 | 4360 | 0.72 | 0.71 | 0.69 | 0.64 | 1.00E-06 |
| root_1 | 28 | 4380 | 0.74 | 0.72 | 0.69 | 0.65 | 1.00E-06 |
| root_1 | 28 | 4400 | 0.70 | 0.72 | 0.71 | 0.65 | 1.00E-06 |
| root_1 | 28 | 4420 | 0.70 | 0.72 | 0.63 | 0.64 | 1.00E-06 |
| root_1 | 28 | 4440 | 0.76 | 0.72 | 0.76 | 0.65 | 1.00E-06 |
| root_1 | 28 | 4450 | 0.67 | NA | 0.85 | NA | 1.00E-06 |
| root_1 | 28 | 4460 | 0.72 | 0.73 | 0.54 | 0.64 | 1.00E-06 |
| root_1 | 28 | 4480 | 0.63 | 0.73 | 0.85 | 0.65 | 1.00E-06 |
| root_1 | 28 | 4500 | 0.64 | 0.72 | 0.80 | 0.64 | 1.00E-06 |
| root_1 | 29 | 4520 | 0.67 | 0.73 | 0.75 | 0.64 | 1.00E-06 |
| root_1 | 29 | 4540 | 0.63 | 0.72 | 0.70 | 0.65 | 1.00E-06 |
| root_1 | 29 | 4550 | 0.59 | NA | 0.71 | NA | 1.00E-06 |
| root_1 | 29 | 4560 | 0.79 | 0.72 | 0.58 | 0.64 | 1.00E-06 |
| root_1 | 29 | 4580 | 0.65 | 0.72 | 0.84 | 0.64 | 1.00E-06 |
| root_1 | 29 | 4600 | 0.66 | 0.71 | 0.87 | 0.64 | 1.00E-06 |
| root_1 | 29 | 4620 | 0.66 | 0.71 | 0.66 | 0.65 | 1.00E-06 |
| root_1 | 29 | 4640 | 0.61 | 0.72 | 0.71 | 0.63 | 1.00E-06 |
| root_1 | 29 | 4650 | 0.78 | NA | 0.47 | NA | 1.00E-06 |
| root_1 | 29 | 4660 | 0.73 | 0.73 | 0.57 | 0.63 | 1.00E-06 |
| root_1 | 30 | 4680 | 0.67 | 0.72 | 0.65 | 0.64 | 1.00E-06 |
| root_1 | 30 | 4700 | 0.67 | 0.73 | 0.62 | 0.63 | 1.00E-06 |
| root_1 | 30 | 4720 | 0.67 | 0.73 | 0.72 | 0.63 | 1.00E-06 |
| root_1 | 30 | 4740 | 0.59 | 0.72 | 0.94 | 0.63 | 1.00E-06 |
| root_1 | 30 | 4750 | 0.69 | NA | 0.66 | NA | 1.00E-06 |
| root_1 | 30 | 4760 | 0.53 | 0.72 | 0.95 | 0.63 | 1.00E-06 |
| root_1 | 30 | 4780 | 0.69 | 0.73 | 0.92 | 0.64 | 1.00E-06 |
| root_1 | 30 | 4800 | 0.69 | 0.73 | 0.51 | 0.62 | 1.00E-06 |
| root_1 | 30 | 4820 | 0.67 | 0.73 | 0.77 | 0.63 | 1.00E-06 |
| root_1 | 31 | 4840 | 0.75 | 0.72 | 0.70 | 0.63 | 1.00E-07 |
| root_1 | 31 | 4850 | 0.72 | NA | 0.74 | NA | 1.00E-07 |
| root_1 | 31 | 4860 | 0.81 | 0.72 | 0.46 | 0.63 | 1.00E-07 |
| root_1 | 31 | 4880 | 0.70 | 0.73 | 0.70 | 0.63 | 1.00E-07 |
| root_1 | 31 | 4900 | 0.64 | 0.75 | 0.77 | 0.63 | 1.00E-07 |
| root_1 | 31 | 4920 | 0.63 | 0.73 | 0.82 | 0.63 | 1.00E-07 |
| root_1 | 31 | 4940 | 0.72 | 0.73 | 0.62 | 0.64 | 1.00E-07 |
| root_1 | 31 | 4950 | 0.69 | NA | 0.73 | NA | 1.00E-07 |
| root_1 | 31 | 4960 | 0.64 | 0.73 | 0.87 | 0.63 | 1.00E-07 |
| root_1 | 31 | 4980 | 0.78 | 0.73 | 0.50 | 0.62 | 1.00E-07 |
| root_1 | 32 | 5000 | 0.66 | 0.72 | 0.79 | 0.63 | 1.00E-07 |
| root_1 | 32 | 5020 | 0.69 | 0.74 | 0.78 | 0.63 | 1.00E-07 |
| root_1 | 32 | 5040 | 0.80 | 0.73 | 0.54 | 0.63 | 1.00E-07 |
| root_1 | 32 | 5050 | 0.69 | NA | 0.88 | NA | 1.00E-07 |
| root_1 | 32 | 5060 | 0.64 | 0.73 | 0.78 | 0.63 | 1.00E-07 |
| root_1 | 32 | 5080 | 0.59 | 0.72 | 0.86 | 0.62 | 1.00E-07 |
| root_1 | 32 | 5100 | 0.69 | 0.73 | 0.79 | 0.63 | 1.00E-07 |
| root_1 | 32 | 5120 | 0.69 | 0.73 | 0.72 | 0.63 | 1.00E-07 |
| root_1 | 32 | 5140 | 0.78 | 0.72 | 0.53 | 0.63 | 1.00E-07 |
| root_1 | 32 | 5150 | 0.64 | NA | 0.60 | NA | 1.00E-07 |
| root_1 | 33 | 5160 | 0.64 | 0.73 | 0.77 | 0.63 | 1.00E-07 |
| root_1 | 33 | 5180 | 0.72 | 0.72 | 0.62 | 0.63 | 1.00E-07 |
| root_1 | 33 | 5200 | 0.70 | 0.72 | 0.60 | 0.63 | 1.00E-07 |
| root_1 | 33 | 5220 | 0.70 | 0.73 | 0.72 | 0.63 | 1.00E-07 |
| root_1 | 33 | 5240 | 0.75 | 0.73 | 0.75 | 0.63 | 1.00E-07 |
| root_1 | 33 | 5250 | 0.66 | NA | 0.85 | NA | 1.00E-07 |
| root_1 | 33 | 5260 | 0.70 | 0.72 | 0.56 | 0.63 | 1.00E-07 |
| root_1 | 33 | 5280 | 0.72 | 0.73 | 0.71 | 0.62 | 1.00E-07 |
| root_1 | 33 | 5300 | 0.61 | 0.73 | 0.75 | 0.63 | 1.00E-07 |
| root_1 | 34 | 5320 | 0.77 | 0.72 | 0.61 | 0.63 | 1.00E-07 |
| root_1 | 34 | 5340 | 0.64 | 0.73 | 0.86 | 0.63 | 1.00E-07 |
| root_1 | 34 | 5350 | 0.58 | NA | 0.94 | NA | 1.00E-07 |
| root_1 | 34 | 5360 | 0.75 | 0.73 | 0.57 | 0.62 | 1.00E-07 |
| root_1 | 34 | 5380 | 0.73 | 0.73 | 0.83 | 0.63 | 1.00E-07 |
| root_1 | 34 | 5400 | 0.73 | 0.74 | 0.62 | 0.62 | 1.00E-07 |
| root_1 | 34 | 5420 | 0.66 | 0.73 | 0.80 | 0.63 | 1.00E-07 |
| root_1 | 34 | 5440 | 0.73 | 0.73 | 0.65 | 0.63 | 1.00E-07 |
| root_1 | 34 | 5450 | 0.69 | NA | 0.56 | NA | 1.00E-07 |
| root_1 | 34 | 5460 | 0.70 | 0.73 | 0.74 | 0.62 | 1.00E-07 |
| root_1 | 35 | 5480 | 0.70 | 0.73 | 0.62 | 0.62 | 1.00E-07 |
| root_1 | 35 | 5500 | 0.61 | 0.73 | 0.79 | 0.62 | 1.00E-07 |
| root_1 | 35 | 5520 | 0.80 | 0.74 | 0.53 | 0.63 | 1.00E-07 |
| root_1 | 35 | 5540 | 0.66 | 0.73 | 0.75 | 0.63 | 1.00E-07 |
| root_1 | 35 | 5550 | 0.66 | NA | 0.70 | NA | 1.00E-07 |
| root_1 | 35 | 5560 | 0.75 | 0.73 | 0.52 | 0.62 | 1.00E-07 |
| root_1 | 35 | 5580 | 0.69 | 0.72 | 0.73 | 0.63 | 1.00E-07 |
| root_1 | 35 | 5600 | 0.77 | 0.73 | 0.55 | 0.63 | 1.00E-07 |
| root_1 | 35 | 5620 | 0.67 | 0.73 | 0.71 | 0.63 | 1.00E-07 |
| root_1 | 36 | 5640 | 0.66 | 0.73 | 0.71 | 0.63 | 1.00E-07 |
| root_1 | 36 | 5650 | 0.72 | NA | 0.60 | NA | 1.00E-07 |
| root_1 | 36 | 5660 | 0.61 | 0.72 | 0.89 | 0.64 | 1.00E-07 |
| root_1 | 36 | 5680 | 0.67 | 0.72 | 0.91 | 0.63 | 1.00E-07 |
| root_1 | 36 | 5700 | 0.67 | 0.73 | 0.85 | 0.63 | 1.00E-07 |
| root_1 | 36 | 5720 | 0.75 | 0.72 | 0.59 | 0.63 | 1.00E-07 |
| root_1 | 36 | 5740 | 0.75 | 0.73 | 0.66 | 0.63 | 1.00E-07 |
| root_1 | 36 | 5750 | 0.64 | NA | 0.71 | NA | 1.00E-07 |
| root_1 | 36 | 5760 | 0.77 | 0.72 | 0.63 | 0.62 | 1.00E-07 |
| root_1 | 36 | 5780 | 0.69 | 0.73 | 0.72 | 0.63 | 1.00E-07 |
| root_1 | 37 | 5800 | 0.72 | 0.73 | 0.73 | 0.63 | 1.00E-07 |
| root_1 | 37 | 5820 | 0.64 | 0.74 | 0.63 | 0.62 | 1.00E-07 |
| root_1 | 37 | 5840 | 0.77 | 0.72 | 0.54 | 0.64 | 1.00E-07 |
| root_1 | 37 | 5850 | 0.64 | NA | 0.74 | NA | 1.00E-07 |
| root_1 | 37 | 5860 | 0.73 | 0.73 | 0.87 | 0.62 | 1.00E-07 |
| root_1 | 37 | 5880 | 0.67 | 0.72 | 0.81 | 0.63 | 1.00E-07 |
| root_1 | 37 | 5900 | 0.61 | 0.73 | 0.81 | 0.63 | 1.00E-07 |
| root_2 | 1 | 1 | 0.35 | 0.38 | 1.58 | 1.57 | 1.00E-04 |
| root_2 | 1 | 10 | 0.48 | 0.52 | 2.05 | 1.75 | 1.00E-04 |
| root_2 | 1 | 20 | 0.49 | 0.53 | 1.88 | 1.89 | 1.00E-04 |
| root_2 | 1 | 30 | 0.54 | 0.57 | 2.19 | 1.72 | 1.00E-04 |
| root_2 | 1 | 40 | 0.53 | 0.55 | 2.00 | 2.08 | 1.00E-04 |
| root_2 | 1 | 50 | 0.54 | 0.59 | 1.95 | 1.71 | 1.00E-04 |
| root_2 | 1 | 60 | 0.51 | 0.60 | 2.37 | 1.73 | 1.00E-04 |
| root_2 | 1 | 70 | 0.54 | 0.58 | 1.88 | 1.57 | 1.00E-04 |
| root_2 | 1 | 80 | 0.58 | 0.60 | 1.83 | 1.71 | 1.00E-04 |
| root_2 | 2 | 90 | 0.55 | 0.61 | 1.77 | 1.46 | 1.00E-04 |
| root_2 | 2 | 100 | 0.64 | 0.59 | 1.56 | 1.74 | 1.00E-04 |
| root_2 | 2 | 110 | 0.57 | 0.64 | 1.56 | 1.35 | 1.00E-04 |
| root_2 | 2 | 120 | 0.60 | 0.68 | 1.72 | 1.21 | 1.00E-04 |
| root_2 | 2 | 130 | 0.57 | 0.65 | 1.81 | 1.46 | 1.00E-04 |
| root_2 | 2 | 140 | 0.66 | 0.66 | 1.45 | 1.31 | 1.00E-04 |
| root_2 | 2 | 150 | 0.63 | 0.69 | 1.38 | 1.00 | 1.00E-04 |
| root_2 | 2 | 160 | 0.67 | 0.70 | 1.10 | 1.05 | 1.00E-04 |
| root_2 | 3 | 170 | 0.66 | 0.70 | 1.17 | 1.04 | 1.00E-04 |
| root_2 | 3 | 180 | 0.68 | 0.73 | 1.24 | 1.01 | 1.00E-04 |
| root_2 | 3 | 190 | 0.71 | 0.72 | 1.01 | 0.97 | 1.00E-04 |
| root_2 | 3 | 200 | 0.71 | 0.72 | 1.16 | 0.96 | 1.00E-04 |
| root_2 | 3 | 210 | 0.70 | 0.73 | 1.05 | 0.94 | 1.00E-04 |
| root_2 | 3 | 220 | 0.71 | 0.73 | 1.00 | 0.90 | 1.00E-04 |
| root_2 | 3 | 230 | 0.70 | 0.72 | 1.08 | 0.94 | 1.00E-04 |
| root_2 | 3 | 240 | 0.69 | 0.71 | 1.05 | 0.98 | 1.00E-04 |
| root_2 | 4 | 250 | 0.64 | 0.72 | 1.30 | 0.91 | 1.00E-04 |
| root_2 | 4 | 260 | 0.75 | 0.68 | 0.79 | 1.10 | 1.00E-04 |
| root_2 | 4 | 270 | 0.73 | 0.70 | 0.95 | 0.98 | 1.00E-04 |
| root_2 | 4 | 280 | 0.68 | 0.74 | 1.03 | 0.90 | 1.00E-04 |
| root_2 | 4 | 290 | 0.71 | 0.76 | 1.01 | 0.89 | 1.00E-04 |
| root_2 | 4 | 300 | 0.64 | 0.73 | 1.19 | 0.95 | 1.00E-04 |
| root_2 | 4 | 310 | 0.68 | 0.72 | 1.10 | 0.95 | 1.00E-04 |
| root_2 | 4 | 320 | 0.69 | 0.74 | 1.15 | 0.84 | 1.00E-04 |
| root_2 | 5 | 330 | 0.68 | 0.72 | 1.00 | 0.89 | 1.00E-04 |
| root_2 | 5 | 340 | 0.66 | 0.75 | 1.22 | 0.86 | 1.00E-04 |
| root_2 | 5 | 350 | 0.70 | 0.74 | 1.01 | 0.92 | 1.00E-04 |
| root_2 | 5 | 360 | 0.72 | 0.72 | 0.98 | 0.95 | 1.00E-04 |
| root_2 | 5 | 370 | 0.74 | 0.75 | 1.00 | 0.89 | 1.00E-04 |
| root_2 | 5 | 380 | 0.73 | 0.75 | 0.99 | 0.90 | 1.00E-04 |
| root_2 | 5 | 390 | 0.73 | 0.76 | 1.07 | 0.86 | 1.00E-04 |
| root_2 | 5 | 400 | 0.72 | 0.77 | 1.00 | 0.75 | 1.00E-04 |
| root_2 | 6 | 410 | 0.72 | 0.76 | 1.00 | 0.79 | 1.00E-04 |
| root_2 | 6 | 420 | 0.81 | 0.78 | 0.63 | 0.77 | 1.00E-04 |
| root_2 | 6 | 430 | 0.70 | 0.76 | 0.98 | 0.81 | 1.00E-04 |
| root_2 | 6 | 440 | 0.76 | 0.76 | 0.83 | 0.78 | 1.00E-04 |
| root_2 | 6 | 450 | 0.72 | 0.75 | 0.97 | 0.89 | 1.00E-04 |
| root_2 | 6 | 460 | 0.70 | 0.74 | 1.05 | 0.88 | 1.00E-04 |
| root_2 | 6 | 470 | 0.71 | 0.76 | 1.13 | 0.89 | 1.00E-04 |
| root_2 | 6 | 480 | 0.73 | 0.77 | 1.02 | 0.88 | 1.00E-04 |
| root_2 | 7 | 490 | 0.75 | 0.78 | 1.00 | 0.82 | 1.00E-04 |
| root_2 | 7 | 500 | 0.77 | 0.75 | 0.92 | 0.87 | 1.00E-04 |
| root_2 | 7 | 510 | 0.75 | 0.78 | 0.91 | 0.74 | 1.00E-04 |
| root_2 | 7 | 520 | 0.76 | 0.77 | 0.87 | 0.86 | 1.00E-04 |
| root_2 | 7 | 530 | 0.69 | 0.75 | 1.19 | 0.91 | 1.00E-04 |
| root_2 | 7 | 540 | 0.73 | 0.76 | 0.87 | 0.85 | 1.00E-04 |
| root_2 | 7 | 550 | 0.70 | 0.74 | 0.88 | 0.84 | 1.00E-04 |
| root_2 | 7 | 560 | 0.73 | 0.76 | 0.88 | 0.74 | 1.00E-04 |
| root_2 | 8 | 570 | 0.73 | 0.76 | 0.74 | 0.80 | 1.00E-04 |
| root_2 | 8 | 580 | 0.69 | 0.77 | 0.89 | 0.72 | 1.00E-04 |
| root_2 | 8 | 590 | 0.77 | 0.77 | 0.75 | 0.76 | 1.00E-04 |
| root_2 | 8 | 600 | 0.70 | 0.77 | 0.96 | 0.76 | 1.00E-04 |
| root_2 | 8 | 610 | 0.70 | 0.77 | 1.18 | 0.77 | 1.00E-04 |
| root_2 | 8 | 620 | 0.76 | 0.77 | 0.81 | 0.72 | 1.00E-04 |
| root_2 | 8 | 630 | 0.75 | 0.79 | 0.81 | 0.69 | 1.00E-04 |
| root_2 | 8 | 640 | 0.76 | 0.79 | 0.91 | 0.71 | 1.00E-04 |
| root_2 | 9 | 650 | 0.73 | 0.79 | 0.74 | 0.70 | 1.00E-04 |
| root_2 | 9 | 660 | 0.77 | 0.78 | 0.78 | 0.69 | 1.00E-04 |
| root_2 | 9 | 670 | 0.71 | 0.78 | 0.92 | 0.75 | 1.00E-04 |
| root_2 | 9 | 680 | 0.78 | 0.79 | 0.82 | 0.72 | 1.00E-04 |
| root_2 | 9 | 690 | 0.77 | 0.80 | 0.77 | 0.64 | 1.00E-04 |
| root_2 | 9 | 700 | 0.72 | 0.77 | 0.78 | 0.70 | 1.00E-04 |
| root_2 | 9 | 710 | 0.78 | 0.74 | 0.68 | 0.79 | 1.00E-04 |
| root_2 | 9 | 720 | 0.80 | 0.78 | 0.63 | 0.65 | 1.00E-04 |
| root_2 | 10 | 730 | 0.82 | 0.78 | 0.62 | 0.69 | 1.00E-04 |
| root_2 | 10 | 740 | 0.73 | 0.77 | 0.89 | 0.70 | 1.00E-04 |
| root_2 | 10 | 750 | 0.74 | 0.76 | 0.79 | 0.79 | 1.00E-04 |
| root_2 | 10 | 760 | 0.79 | 0.77 | 0.72 | 0.71 | 1.00E-04 |
| root_2 | 10 | 770 | 0.77 | 0.78 | 0.73 | 0.68 | 1.00E-04 |
| root_2 | 10 | 780 | 0.73 | 0.76 | 0.83 | 0.78 | 1.00E-04 |
| root_2 | 10 | 790 | 0.80 | 0.79 | 0.65 | 0.69 | 1.00E-04 |
| root_2 | 10 | 800 | 0.80 | 0.81 | 0.72 | 0.60 | 1.00E-04 |
| root_2 | 10 | 810 | 0.73 | 0.78 | 0.89 | 0.70 | 1.00E-04 |
| root_2 | 11 | 820 | 0.73 | 0.75 | 1.05 | 0.98 | 1.00E-05 |
| root_2 | 11 | 830 | 0.76 | 0.79 | 0.81 | 0.72 | 1.00E-05 |
| root_2 | 11 | 840 | 0.74 | 0.79 | 0.87 | 0.63 | 1.00E-05 |
| root_2 | 11 | 850 | 0.78 | 0.81 | 0.66 | 0.59 | 1.00E-05 |
| root_2 | 11 | 860 | 0.74 | 0.81 | 0.86 | 0.56 | 1.00E-05 |
| root_2 | 11 | 870 | 0.79 | 0.82 | 0.59 | 0.54 | 1.00E-05 |
| root_2 | 11 | 880 | 0.78 | 0.82 | 0.66 | 0.53 | 1.00E-05 |
| root_2 | 11 | 890 | 0.80 | 0.82 | 0.61 | 0.51 | 1.00E-05 |
| root_2 | 12 | 900 | 0.79 | 0.83 | 0.64 | 0.51 | 1.00E-05 |
| root_2 | 12 | 910 | 0.84 | 0.82 | 0.52 | 0.51 | 1.00E-05 |
| root_2 | 12 | 920 | 0.79 | 0.82 | 0.59 | 0.51 | 1.00E-05 |
| root_2 | 12 | 930 | 0.78 | 0.82 | 0.61 | 0.50 | 1.00E-05 |
| root_2 | 12 | 940 | 0.79 | 0.82 | 0.60 | 0.50 | 1.00E-05 |
| root_2 | 12 | 950 | 0.79 | 0.82 | 0.57 | 0.50 | 1.00E-05 |
| root_2 | 12 | 960 | 0.79 | 0.82 | 0.61 | 0.50 | 1.00E-05 |
| root_2 | 12 | 970 | 0.78 | 0.83 | 0.63 | 0.48 | 1.00E-05 |
| root_2 | 13 | 980 | 0.83 | 0.83 | 0.56 | 0.48 | 1.00E-05 |
| root_2 | 13 | 990 | 0.82 | 0.83 | 0.49 | 0.48 | 1.00E-05 |
| root_2 | 13 | 1000 | 0.83 | 0.83 | 0.42 | 0.47 | 1.00E-05 |
| root_2 | 13 | 1010 | 0.80 | 0.82 | 0.46 | 0.49 | 1.00E-05 |
| root_2 | 13 | 1020 | 0.80 | 0.83 | 0.57 | 0.48 | 1.00E-05 |
| root_2 | 13 | 1030 | 0.80 | 0.83 | 0.55 | 0.49 | 1.00E-05 |
| root_2 | 13 | 1040 | 0.79 | 0.82 | 0.61 | 0.50 | 1.00E-05 |
| root_2 | 13 | 1050 | 0.78 | 0.83 | 0.71 | 0.48 | 1.00E-05 |
| root_2 | 14 | 1060 | 0.82 | 0.84 | 0.53 | 0.47 | 1.00E-05 |
| root_2 | 14 | 1070 | 0.80 | 0.83 | 0.66 | 0.48 | 1.00E-05 |
| root_2 | 14 | 1080 | 0.81 | 0.83 | 0.53 | 0.48 | 1.00E-05 |
| root_2 | 14 | 1090 | 0.77 | 0.83 | 0.58 | 0.46 | 1.00E-05 |
| root_2 | 14 | 1100 | 0.78 | 0.83 | 0.61 | 0.48 | 1.00E-05 |
| root_2 | 14 | 1110 | 0.78 | 0.83 | 0.61 | 0.48 | 1.00E-05 |
| root_2 | 14 | 1120 | 0.81 | 0.83 | 0.50 | 0.48 | 1.00E-05 |
| root_2 | 14 | 1130 | 0.77 | 0.83 | 0.60 | 0.47 | 1.00E-05 |
| root_2 | 15 | 1140 | 0.78 | 0.83 | 0.63 | 0.48 | 1.00E-05 |
| root_2 | 15 | 1150 | 0.78 | 0.82 | 0.67 | 0.48 | 1.00E-05 |
| root_2 | 15 | 1160 | 0.80 | 0.83 | 0.50 | 0.48 | 1.00E-05 |
| root_2 | 15 | 1170 | 0.78 | 0.83 | 0.53 | 0.47 | 1.00E-05 |
| root_2 | 15 | 1180 | 0.81 | 0.83 | 0.56 | 0.46 | 1.00E-05 |
| root_2 | 15 | 1190 | 0.77 | 0.83 | 0.54 | 0.47 | 1.00E-05 |
| root_2 | 15 | 1200 | 0.82 | 0.83 | 0.45 | 0.47 | 1.00E-05 |
| root_2 | 15 | 1210 | 0.82 | 0.83 | 0.54 | 0.48 | 1.00E-05 |
| root_2 | 16 | 1220 | 0.81 | 0.84 | 0.47 | 0.45 | 1.00E-05 |
| root_2 | 16 | 1230 | 0.74 | 0.83 | 0.75 | 0.47 | 1.00E-05 |
| root_2 | 16 | 1240 | 0.79 | 0.84 | 0.60 | 0.45 | 1.00E-05 |
| root_2 | 16 | 1250 | 0.85 | 0.84 | 0.50 | 0.46 | 1.00E-05 |
| root_2 | 16 | 1260 | 0.84 | 0.83 | 0.45 | 0.46 | 1.00E-05 |
| root_2 | 16 | 1270 | 0.76 | 0.83 | 0.65 | 0.47 | 1.00E-05 |
| root_2 | 16 | 1280 | 0.77 | 0.83 | 0.62 | 0.47 | 1.00E-05 |
| root_2 | 16 | 1290 | 0.81 | 0.84 | 0.47 | 0.45 | 1.00E-05 |
| root_2 | 17 | 1300 | 0.80 | 0.83 | 0.48 | 0.45 | 1.00E-05 |
| root_2 | 17 | 1310 | 0.82 | 0.84 | 0.53 | 0.45 | 1.00E-05 |
| root_2 | 17 | 1320 | 0.81 | 0.84 | 0.48 | 0.46 | 1.00E-05 |
| root_2 | 17 | 1330 | 0.86 | 0.84 | 0.41 | 0.45 | 1.00E-05 |
| root_2 | 17 | 1340 | 0.81 | 0.84 | 0.50 | 0.44 | 1.00E-05 |
| root_2 | 17 | 1350 | 0.81 | 0.83 | 0.52 | 0.47 | 1.00E-05 |
| root_2 | 17 | 1360 | 0.78 | 0.82 | 0.66 | 0.45 | 1.00E-05 |
| root_2 | 17 | 1370 | 0.75 | 0.83 | 0.66 | 0.45 | 1.00E-05 |
| root_2 | 18 | 1380 | 0.85 | 0.83 | 0.45 | 0.46 | 1.00E-05 |
| root_2 | 18 | 1390 | 0.83 | 0.83 | 0.47 | 0.45 | 1.00E-05 |
| root_2 | 18 | 1400 | 0.78 | 0.84 | 0.52 | 0.43 | 1.00E-05 |
| root_2 | 18 | 1410 | 0.82 | 0.84 | 0.44 | 0.43 | 1.00E-05 |
| root_2 | 18 | 1420 | 0.85 | 0.84 | 0.41 | 0.45 | 1.00E-05 |
| root_2 | 18 | 1430 | 0.82 | 0.84 | 0.52 | 0.44 | 1.00E-05 |
| root_2 | 18 | 1440 | 0.78 | 0.85 | 0.55 | 0.42 | 1.00E-05 |
| root_2 | 18 | 1450 | 0.75 | 0.84 | 0.58 | 0.42 | 1.00E-05 |
| root_2 | 19 | 1460 | 0.83 | 0.85 | 0.42 | 0.43 | 1.00E-05 |
| root_2 | 19 | 1470 | 0.78 | 0.84 | 0.53 | 0.43 | 1.00E-05 |
| root_2 | 19 | 1480 | 0.82 | 0.84 | 0.49 | 0.42 | 1.00E-05 |
| root_2 | 19 | 1490 | 0.83 | 0.83 | 0.42 | 0.44 | 1.00E-05 |
| root_2 | 19 | 1500 | 0.83 | 0.84 | 0.51 | 0.43 | 1.00E-05 |
| root_2 | 19 | 1510 | 0.80 | 0.84 | 0.53 | 0.44 | 1.00E-05 |
| root_2 | 19 | 1520 | 0.82 | 0.83 | 0.53 | 0.45 | 1.00E-05 |
| root_2 | 19 | 1530 | 0.83 | 0.84 | 0.42 | 0.42 | 1.00E-05 |
| root_2 | 20 | 1540 | 0.79 | 0.85 | 0.49 | 0.42 | 1.00E-05 |
| root_2 | 20 | 1550 | 0.84 | 0.85 | 0.39 | 0.41 | 1.00E-05 |
| root_2 | 20 | 1560 | 0.78 | 0.85 | 0.57 | 0.43 | 1.00E-05 |
| root_2 | 20 | 1570 | 0.81 | 0.85 | 0.53 | 0.41 | 1.00E-05 |
| root_2 | 20 | 1580 | 0.81 | 0.84 | 0.45 | 0.43 | 1.00E-05 |
| root_2 | 20 | 1590 | 0.82 | 0.83 | 0.53 | 0.42 | 1.00E-05 |
| root_2 | 20 | 1600 | 0.77 | 0.83 | 0.59 | 0.43 | 1.00E-05 |
| root_2 | 20 | 1610 | 0.83 | 0.84 | 0.48 | 0.43 | 1.00E-05 |
| root_2 | 20 | 1620 | 0.78 | 0.84 | 0.56 | 0.44 | 1.00E-05 |
| root_2 | 21 | 1630 | 0.84 | 0.84 | 0.43 | 0.43 | 1.00E-06 |
| root_2 | 21 | 1640 | 0.84 | 0.84 | 0.46 | 0.43 | 1.00E-06 |
| root_2 | 21 | 1650 | 0.82 | 0.84 | 0.54 | 0.42 | 1.00E-06 |
| root_2 | 21 | 1660 | 0.84 | 0.85 | 0.45 | 0.42 | 1.00E-06 |
| root_2 | 21 | 1670 | 0.77 | 0.84 | 0.46 | 0.41 | 1.00E-06 |
| root_2 | 21 | 1680 | 0.84 | 0.85 | 0.41 | 0.41 | 1.00E-06 |
| root_2 | 21 | 1690 | 0.85 | 0.84 | 0.49 | 0.42 | 1.00E-06 |
| root_2 | 21 | 1700 | 0.80 | 0.85 | 0.58 | 0.41 | 1.00E-06 |
| root_2 | 22 | 1710 | 0.88 | 0.85 | 0.35 | 0.41 | 1.00E-06 |
| root_2 | 22 | 1720 | 0.80 | 0.85 | 0.49 | 0.40 | 1.00E-06 |
| root_2 | 22 | 1730 | 0.82 | 0.84 | 0.43 | 0.42 | 1.00E-06 |
| root_2 | 22 | 1740 | 0.80 | 0.85 | 0.47 | 0.40 | 1.00E-06 |
| root_2 | 22 | 1750 | 0.80 | 0.85 | 0.46 | 0.41 | 1.00E-06 |
| root_2 | 22 | 1760 | 0.84 | 0.85 | 0.42 | 0.41 | 1.00E-06 |
| root_2 | 22 | 1770 | 0.73 | 0.84 | 0.76 | 0.42 | 1.00E-06 |
| root_2 | 22 | 1780 | 0.81 | 0.84 | 0.58 | 0.42 | 1.00E-06 |
| root_2 | 23 | 1790 | 0.90 | 0.84 | 0.27 | 0.42 | 1.00E-06 |
| root_2 | 23 | 1800 | 0.82 | 0.85 | 0.45 | 0.41 | 1.00E-06 |
| root_2 | 23 | 1810 | 0.82 | 0.85 | 0.49 | 0.41 | 1.00E-06 |
| root_2 | 23 | 1820 | 0.86 | 0.85 | 0.38 | 0.39 | 1.00E-06 |
| root_2 | 23 | 1830 | 0.78 | 0.85 | 0.57 | 0.40 | 1.00E-06 |
| root_2 | 23 | 1840 | 0.81 | 0.84 | 0.43 | 0.41 | 1.00E-06 |
| root_2 | 23 | 1850 | 0.86 | 0.86 | 0.40 | 0.38 | 1.00E-06 |
| root_2 | 23 | 1860 | 0.82 | 0.85 | 0.41 | 0.39 | 1.00E-06 |
| root_2 | 24 | 1870 | 0.79 | 0.85 | 0.49 | 0.39 | 1.00E-06 |
| root_2 | 24 | 1880 | 0.78 | 0.85 | 0.51 | 0.39 | 1.00E-06 |
| root_2 | 24 | 1890 | 0.78 | 0.85 | 0.74 | 0.39 | 1.00E-06 |
| root_2 | 24 | 1900 | 0.80 | 0.84 | 0.46 | 0.41 | 1.00E-06 |
| root_2 | 24 | 1910 | 0.78 | 0.85 | 0.59 | 0.39 | 1.00E-06 |
| root_2 | 24 | 1920 | 0.86 | 0.84 | 0.45 | 0.39 | 1.00E-06 |
| root_2 | 24 | 1930 | 0.85 | 0.85 | 0.45 | 0.40 | 1.00E-06 |
| root_2 | 24 | 1940 | 0.77 | 0.85 | 0.53 | 0.41 | 1.00E-06 |
| root_2 | 25 | 1950 | 0.80 | 0.85 | 0.48 | 0.39 | 1.00E-06 |
| root_2 | 25 | 1960 | 0.80 | 0.84 | 0.53 | 0.40 | 1.00E-06 |
| root_2 | 25 | 1970 | 0.81 | 0.84 | 0.49 | 0.39 | 1.00E-06 |
| root_2 | 25 | 1980 | 0.82 | 0.84 | 0.40 | 0.40 | 1.00E-06 |
| root_2 | 25 | 1990 | 0.83 | 0.85 | 0.46 | 0.41 | 1.00E-06 |
| root_2 | 25 | 2000 | 0.82 | 0.84 | 0.39 | 0.39 | 1.00E-06 |
| root_2 | 25 | 2010 | 0.83 | 0.85 | 0.45 | 0.40 | 1.00E-06 |
| root_2 | 25 | 2020 | 0.83 | 0.85 | 0.42 | 0.40 | 1.00E-06 |
| root_2 | 26 | 2030 | 0.89 | 0.86 | 0.37 | 0.38 | 1.00E-06 |
| root_2 | 26 | 2040 | 0.91 | 0.85 | 0.29 | 0.39 | 1.00E-06 |
| root_2 | 26 | 2050 | 0.81 | 0.86 | 0.49 | 0.38 | 1.00E-06 |
| root_2 | 26 | 2060 | 0.86 | 0.86 | 0.42 | 0.39 | 1.00E-06 |
| root_2 | 26 | 2070 | 0.78 | 0.86 | 0.42 | 0.36 | 1.00E-06 |
| root_2 | 26 | 2080 | 0.78 | 0.83 | 0.51 | 0.41 | 1.00E-06 |
| root_2 | 26 | 2090 | 0.84 | 0.85 | 0.38 | 0.40 | 1.00E-06 |
| root_2 | 26 | 2100 | 0.88 | 0.86 | 0.49 | 0.36 | 1.00E-06 |
| root_2 | 27 | 2110 | 0.84 | 0.85 | 0.42 | 0.38 | 1.00E-06 |
| root_2 | 27 | 2120 | 0.81 | 0.84 | 0.37 | 0.39 | 1.00E-06 |
| root_2 | 27 | 2130 | 0.91 | 0.86 | 0.43 | 0.35 | 1.00E-06 |
| root_2 | 27 | 2140 | 0.72 | 0.86 | 0.63 | 0.34 | 1.00E-06 |
| root_2 | 27 | 2150 | 0.91 | 0.86 | 0.39 | 0.34 | 1.00E-06 |
| root_2 | 27 | 2160 | 0.81 | 0.86 | 0.79 | 0.36 | 1.00E-06 |
| root_2 | 27 | 2170 | 0.88 | 0.85 | 0.44 | 0.37 | 1.00E-06 |
| root_2 | 27 | 2180 | 0.81 | 0.84 | 0.45 | 0.38 | 1.00E-06 |
| root_2 | 28 | 2190 | 0.75 | 0.86 | 0.67 | 0.36 | 1.00E-06 |
| root_2 | 28 | 2200 | 0.78 | 0.85 | 0.47 | 0.35 | 1.00E-06 |
| root_2 | 28 | 2210 | 0.88 | 0.86 | 0.33 | 0.36 | 1.00E-06 |
| root_2 | 28 | 2220 | 0.84 | 0.86 | 0.38 | 0.36 | 1.00E-06 |
| root_2 | 28 | 2230 | 0.78 | 0.87 | 0.44 | 0.36 | 1.00E-06 |
| root_2 | 28 | 2240 | 0.84 | 0.85 | 0.49 | 0.37 | 1.00E-06 |
| root_2 | 28 | 2250 | 0.72 | 0.84 | 0.63 | 0.37 | 1.00E-06 |
| root_2 | 28 | 2260 | 0.88 | 0.84 | 0.24 | 0.38 | 1.00E-06 |
| root_2 | 29 | 2270 | 0.91 | 0.86 | 0.31 | 0.37 | 1.00E-06 |
